# An improved approach for fault detection by simultaneous overcoming of high-dimensionality, autocorrelation, and time-variability

**DOI:** 10.1371/journal.pone.0243146

**Published:** 2020-12-17

**Authors:** Nastaran Hajarian, Farzad Movahedi Sobhani, Seyed Jafar Sadjadi

**Affiliations:** 1 Department of Industrial Engineering, Science and Research Branch, Islamic Azad University, Tehran, Iran; 2 Department of Industrial Engineering, Iran University of Science and Technology, Tehran, Iran; Tianjin University, CHINA

## Abstract

The control charts with the Principal Component Analysis (PCA) approach and its extension are among the data-driven methods for process monitoring and the detection of faults. Industrial processing data involves complexities such as high dimensionality, auto-correlation, and non-stationary which may occur simultaneously. An efficient fault detection technique is an approach that is robust against data training, sensitive to all the feasible faults of the process, and agile to the detection of the faults. To date, approaches such as the recursive PCA (RPCA) model and the moving-window PCA (MWPCA) model have been proposed when data is high-dimensional and non-stationary or dynamic PCA (DPCA) model and its extension have been suggested for autocorrelation data. But, using the techniques listed without considering all aspects of the process data increases fault detection indicators such as false alarm rate (FAR), delay time detection (DTD), and confuses the operator or causes adverse consequences. A new PCA monitoring method is proposed in this study, which can simultaneously reduce the impact of high-dimensionality, non-stationary, and autocorrelation properties. This technique utilizes DPCA property to decrease the effect of autocorrelation and adaptive behavior of MWPCA to control non-stationary characteristics. The proposed approach has been tested on the Tennessee Eastman Process (TEP). The findings suggest that the proposed approach is capable of detecting various forms of faults and comparing attempts to improve the detection of fault indicators with other approaches. The empirical application of the proposed approach has been implemented on a turbine exit temperature (TET). The results demonstrate that the proposed approach has detected a real fault successfully.

## 1. Introduction

**Fig 1:**
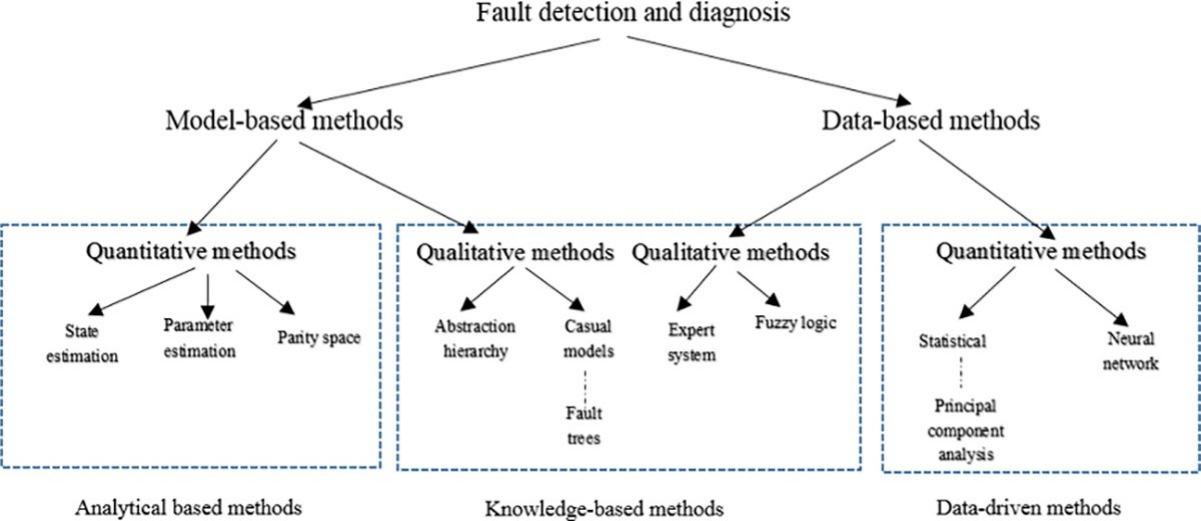
Classification of fault detection and diagnosis method [1].

There are several conceptual approaches in detecting and diagnosing failure, and different researchers, based on their point of view, present various classifications, none of which is comprehensive. Zhang et al. argued that fault detection methods could be either model-based or data-based methods (Fig 1) [1]. However, Chiang et al. believed that there are three fault detecting and diagnosing methods, namely data-driven methods, analytical based methods, and knowledge-based methods [2].

The analytical approach utilizes the first principles to construct mathematical models of the system [3]. The analytical model cannot be applied for large-scale and complex systems. Accordingly, ref. [4], the knowledge-based methods are mostly rule-based expert systems. The failure cases and engineers’ experience are used to formulate the rules. When the detailed mathematical model cannot be reached, and when the number of inputs, outputs, and states of a system is logically limited, the knowledge-based method will yield the best results [4]. To detect faults, the data-driven methods use products’ life-cycle data, which they are not dependent on the first-principles [5, 6]. Hence, data-driven methods can be used for large-scale and complex systems, which are inexpensive as well [3]. Data-driven approaches are chosen upon the availability of product or system data, but the system model is not [7].

As a consequence, a multivariate statistical approach is needed when the number of variables or dimensions of an industrial problem is beyond one value. One of the data-driven multivariate statistical tools is the quality control charts by the principal component analysis approach (PCA) to detect an abnormal behavior [6, 8]. However, in addition to their high dimensionality, data reported to solve sophisticated industrial, health care, IT, or economic problems have characteristics such as autocorrelation and non-stationary nature [9]. Therefore, although it is true that quality control charts based on conventional PCA are capable of managing the high dimensional process and suitable for stationary one, the findings suggested that linear and non-adaptive methods have high false alarm rates because of the incompatibility of this approach with auto-correlated and non-stationary process data [10]. For example, if a static model is implemented on non-stationery data, the model structure is inefficient to estimate the new observation because the mean and variance criteria change over time. Since a formula is used to estimate the new observation, which has the least similarity with the measurement time parameters of the new observation [9].

Concerning the characteristic of autocorrelation, the first choice could be minimizing the effect of autocorrelation by differencing and then using static PCA. But this method causes a major problem in process monitoring. This problem arises when the identification of certain forms of fault, such as step change, emerges. As step change occurs, differencing shows a significant shift in process monitoring, but subsequent faulty observations tend to be under control as they are in relative control of each other [9, 11]. This may result in the operator interpreting this step change as a false alarm.

In line with these problems, many researchers attempted to increase the accuracy of the PCA model. In the literature, various quality control charts have been proposed to overcome autocorrelation or non-stationary problems, including the principal component analysis (PCA).

Ku et al. proposed Dynamic PCA, which is an approach to extend static PCA tools to address autocorrelation in a multivariate process [12]. Rato et al. suggested approaches to improve Dynamic PCA [10, 11]. Ammiche et al. recommended a modified moving window dynamic PCA with a fuzzy logic filter and its application to fault detection. They claimed that this approach could handle the autocorrelation problem and reduce false alarm rates [13].

Li et al. provided a recursive PCA approach to cope with non-stationary problems [14]. The RPCA technique updates the model for ever-increasing data consisting of new samples without discarding the old ones. Theoretically, RPCA, which has been used efficiently for process monitoring, could be simple but has some disadvantages. For instance, as data sets expand, and the model is updated, the speed of compatibility is decreased by growing the size of the data. Furthermore, the forgetting factor cannot be easily selected without prior knowledge of likely fault conditions when older samples are given down-weight. Another approach proposed to cope with the non-stationary problem is moving window PCA (MWPCA) [11, 15]. The MWPCA method can tackle some of the limitations mentioned above by collecting a sufficient number of data points in the time-window that can help build an adaptive process. Specifically, MWPCA removes older samples to choose the new samples representing the current operational process. Meanwhile, researchers have stated the size of the window in MWPCA as an essential parameter. If the appropriate window size is not selected, over-fitting will be observed in the model [16]. Therefore, MWPCA based on the application delay as V-step-ahead prediction was implemented to solve this problem. This method is applied using a model calculated at time t to predict the behavior of the system at time t + V and to detect the possible faults. This step is taken to ensure that the model does not overly adapt to the data, and can detect errors that gradually build up and are recognized as normal observations at any point of time [17]. Ketelaere et al. reviewed the PCA-based statistical process-monitoring methods in terms of time-dependent, high-dimensional data including DPCA, MWPCA, and RPCA [9]. Other researchers proposed various approaches in areas of DPCA, MWPCA, and RPCA for instance, to reduce false alarm rates or combined them with other methods [16, 18–21]. As stated, RPCA and MWPCA are adaptive approaches, indicating that with each new sample correctly identified the monitoring model and control limit would be modified where the normal time-varying information is applied to the monitoring model to distinguish between the normal time-varying and slow ramp fault processes. Nevertheless, Gao et al. introduced another approach to solve non-stationary problems called incremental PCA [22]. This method, unlike the adaptive method, is changeless and introduces a new parameter as an incremental PC (IPC). In this method, the non-stationary problem is extracted by calculating IPCs. Finally, the monitoring model remains unchanged and uses the new statistic called *IT*^2^ to monitor data.

As stated, because the collection sampling is rapid in industrial processes, the data have autocorrelation properties and depending on the situation, the process behavior can change in various modes and parameters such as mean or variance, which makes it non-stationary. As far as DPCA model is concerned, it can resolve auto-correlation, but it has fixed thresholds and when data is non-stationary, indicators such as false alarm rate (FAR), missed detection rate (MDR), and delay time detection (DTD) may suggest that it does not perform well. Also, the methods such as the MWPCA model can dominate the non-stationary problem with adaptive thresholds. Again, precision in the detection of fault is not sufficient because the autocorrelation problem has been overlooked. Increasing the measurement indicators due to the lack of an acceptable system that is consistent with data characteristics causes delay in detecting faults and confounding operators. Based on De Ketelaere [9] suggestion and our investigation, no research has been carried out on the model that can handle high-dimensionality, non-stationary, and autocorrelation, simultaneously. In addition to using DPCA’s property to resolve autocorrelation through data matrix expansion, this research attempts to reduce indicators such as FAR, MDR, and DTD by using the adaptive thresholds function of MWPCA process. In other words, the aim of this article is to present a suitable fault detection approach combined of MWPCA and DPCA properties models that can handle autocorrelation where the time lag of each variable can be different and non-stationary at the same time.

This paper is organized as follows: the background of PCA models and how they are used in fault detection are outlined in Section 2. Section 3 presents the proposed approach, which is a combination of DPCA and MWPCA. In section 4, the proposed approach is implemented on TEP data and turbine exit temperature (TET). Discussion and results are also presented in this section. Finally, the conclusion is given in Section 5.

## 2. Background of PCA

### 2.1. Principal component analysis (PCA)

PCA converts a set of correlated variables into a smaller number of uncorrelated new variables, where the new sample includes the most information from the original data [2, 23, 24]. Let *X*∈*R*^*n*×*m*^ be the original data matrix with n samples and m variables, which can be explained as:

$$X={\left[X_{1}{,X}_{2}\ldots X_{n}\right]}^{T}=\left[\begin{array}{ccc} x_{1,1} & \ldots & x_{1,m}\\ . & \ldots & .\\ x_{n,1} & \ldots & x_{n,m} \end{array}\right]$$ (1)

The first step in PCA is the standardization of the data. After scaling, matrix *X* has zero mean and one-unit variance. Then, the PCA algorithm projects *X* onto a new orthonormal space by the following linear transformation:

$$T=[t_{1},t_{2},\ldots t_{m}]=XP=X[p_{1},p_{2},\ldots p_{m}]$$ (2)

*P* can be calculated from the following eigenvalue problem:

$$C=P\Lambda P^{T}$$ (3)

$$C=\frac{1}{n-1}X^{T}X$$ (4)

Where *C* represents the covariance matrix of *X* calculated by Eq (4) and *Λ* represents a diagonal matrix consisting of the non-negative real eigenvalues in descending order (λ_1_≥λ_2_≥⋯≥λ_m_≥0). The next step is determining principal dimensions or selecting the number of principal components (PCs) according to the distribution of variations in the new coordinate system. Several methods have been proposed for choosing the number of principal components [25, 26]. Cumulative Percent Variance (CPV) is one of these methods which determines the percentage of variance calculated by the first *r* principal components as follows [27]:

$$CPV(r)=\frac{\sum_{i=1}^{r}\lambda_{i}}{trace(R)}$$ (5)

According to eigenvalues that determine how much variation each PC has, *CPV* can be an appropriate criterion to define the count of PCs in a PCA model. After determining the number of PCs, X is decomposed into PC space (*T*_*r*_*P*_*r*_) and residual space (*E*), which is shown in Eq (6).

$$X=T_{r}P_{r}^{T}+E=[t_{1},t_{2},\ldots t_{r}]{\left[p_{1},p_{2},\ldots p_{r}\right]}^{T}+E$$ (6)

PC space explains the information of the system variations, while the residual space describes the information of noise or model error [28].

After constructing a PCA model based on the historical data collected, it is necessary to have the instrument that controls variations. It is possible to plot the multivariate control charts using the Hotelling *T*^2^ and square prediction error (SPE) or *Q* to detect the fault. Determining two orthogonal subspace of the original space can reduce the monitoring into these two variables (*T*^2^ and *Q*) [27].

The major variations and the random noise in the data can be controlled by *T*^2^ and *Q* respectively.

The *T*^2^ statistic can be calculated for each new observation x by:

$$T^{2}=x^{T}P\Lambda_{r}^{-1}P^{T}x$$ (7)

Where *Λ*_*r*_ is the squared matrix constructed by the first *r* rows and columns of *Λ* and, as previously mentioned, *P* represents *r* eigenvectors or columns of *V*. The upper confidence limit for *T*^2^ is acquired using the F-distribution:

$$T_{\alpha }^{2}=\frac{r(n-1)}{n-r}F_{\alpha ,r,n-r}$$ (8)

Where, *r* is the number of the principal components and *n* denotes the number of samples in the data, and *α* is the level of significance. A violation of the threshold would mean that variations of the system are out of control. Another statistic is the squared prediction error (*SPE*) or *Q*, which can monitor the portion of the measurement space related to the lowest *m−r* eigenvalues. Indeed, *Q* statistic is calculated as the sum of squares of residuals.

$$Q=x^{T}(I-PP^{T}]x$$ (9)

Where, *I* is the identity matrix.

The upper confidence limit for the *Q* can be calculated from its approximate distribution:

Qα=θ1[Cα2θ2h02θ1+θ2h0(h0−1)θ12+1]1h0 (10)

$$\theta_{i}=\sum_{j=r+1}^{m}\lambda_{j}^{i}h_{0}=1-\frac{2\theta_{1}\theta_{3}}{3\theta_{2}^{2}}$$

Where, *C*_*α*_ is the value of the normal distribution with the *α* level significance. A violation of the threshold would indicate that an unusual event has occurred causing a change in the covariance structure of the model [29]. Based on the previous description, if *Q* or *T*^2^ statistics are beyond their confidence limits, abnormality is indicated.

**Fig 2:**
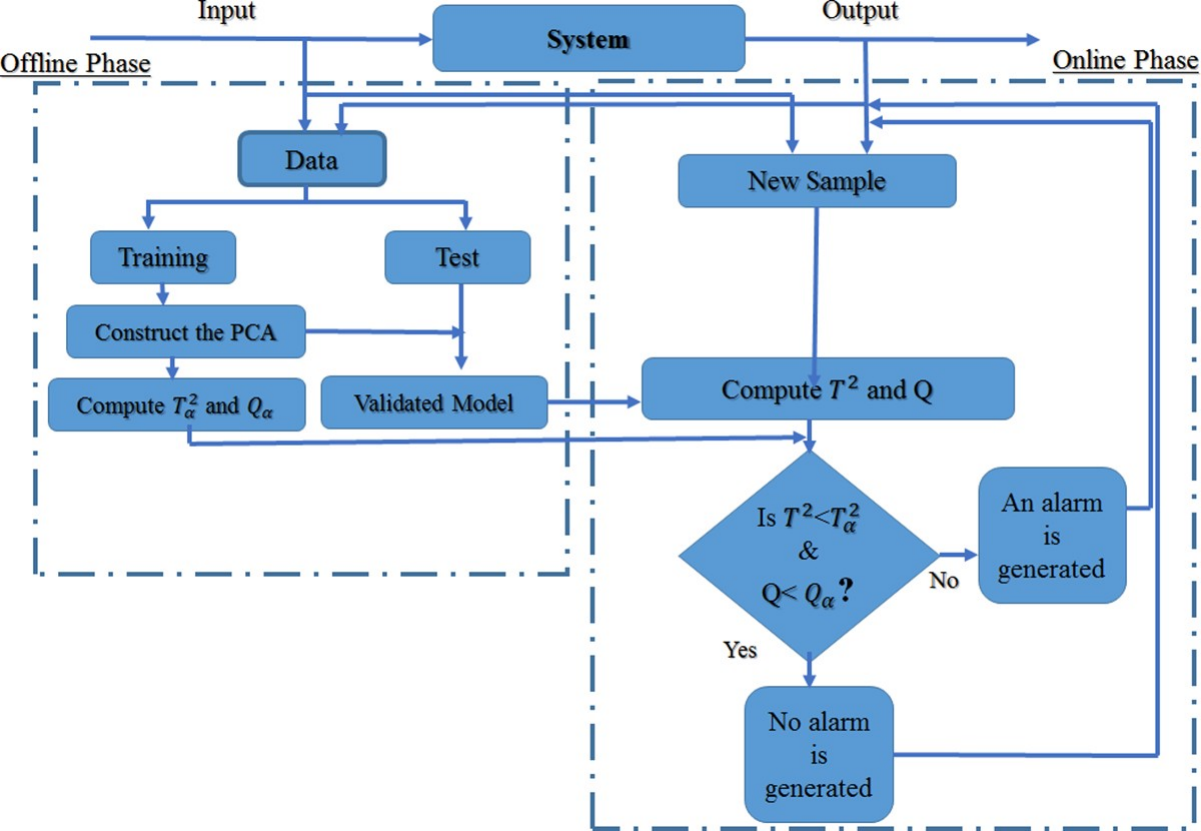
Flowchart of static PCA [30].

In summary, the condition monitoring process with PCA method is demonstrated in Fig 2.

### 2.2. Dynamic principal component analysis

A small sampling period is required for early detection of faults in stream recorded data from fast industrial processes. In other words, the current values of the process variables depend on the past values. Thus, assumption of statistical independency between observations is violated. Hence, the conventional PCA model will not offer a good performance [31]. Ku et al. [12] proposed a dynamic PCA method which added the concept of data dynamics to the PCA model. For this propose, they used time lag shift method and added a necessary number of lags, *l*, to the data. The augmented data matrix is demonstrated further.

$$X_{A}(l)=[\begin{array}{cccccccccccccccc} x_{11} & x_{21} & \cdots & x_{(l+1)1} & | & x_{12} & x_{22} & \cdots & x_{(l+1)2} & | & \cdots & | & x_{1m} & x_{2m} & \cdots & x_{(l+1)m}\\ x_{21} & x_{31} & \cdots & x_{(l+2)1} & | & x_{22} & x_{32} & \cdots & x_{(l+2)2} & | & \cdots & | & x_{2m} & x_{3m} & \cdots & x_{(l+2)m}\\ \vdots & \vdots & \vdots & \vdots & | & \vdots & \vdots & \vdots & \vdots & | & \vdots & | & \vdots & \vdots & \vdots & \vdots \\ x_{(n-l)1} & x_{(n-l+1)1} & \cdots & x_{n1} & | & x_{(n-l)2} & x_{(n-l+1)2} & \cdots & x_{n2} & | & \cdots & | & x_{(n-l)m} & x_{(n-l+1)m} & \cdots & x_{nm} \end{array}]$$ (11)

The DPCA model is constructed by applying PCA model on *X*_*A*_(*l*). The monitoring procedures of the DPCA are the same as the ones of PCA. The important key in the DPCA is selecting the number of lags.

### 2.3. Moving window principal component analysis

The non-stationary property appears since the process parameters, such as the mean or covariance, change over time [11]. To tackle the time-varying issue, several complementary multivariate statistical process monitoring (MSPM) methods have been introduced. To address non-stationary data, three classes of approaches called recursive PCA (RPCA), moving-window PCA (MWPCA) and incremental PCA (IPCA) have been applied to develop PCA methods [22].

As mentioned in the introduction, RPCA, which has been used for process monitoring efficiently might be theoretically simple. However, its implementation might not be accessible due to two main reasons: the ever-growing data set on which the model is updated eventually slows down the speed of adaptation as the data size increases. RPCA also consists of older data that are unrepresentative of the time-varying process. Forgetting factor cannot be easily selected without a priori knowledge of likely fault conditions when given to down-weight older samples [15].

The MWPCA method can tackle some of the aforementioned limitations by collecting a sufficient number of data points in the time-window, which can help build an adaptive process. Specifically, moving window principal component analysis (MWPCA) removes older samples to choose the new ones representing the current operation process. Hence, for window size K, the data matrix at time *k* is *X_k_* = ($x_{k-K+1},x_{k-K+2}$, *…*,*x*_*k*_*)* and, at time *k + 1*, it is *X*_*k*+1_ = ($x_{k-K+2},x_{k-K+3}$, …, *x*_*k*+1_). The observations in the new window can be used to obtain the updated ${\bar{x}}_{k+1}$ and *s*_*k*+1_ [32]. The MWPCA algorithm can be summarized as follows:

**Offline step:**

1. Implementation of a conventional PCA model on training data (computing the loading vectors, number of principal components, and control limits of the monitoring indexes, *T*^2^ and *Q* statistics).
2. Model validation, which is performed with test data.

**Online step:**

1. Select a new online sample. Normalize the sample with the means and variances of the training dataset.
2. Calculate the monitoring indices for this new sample (*T*^2^ and *Q* statistics).
3. Compare the monitoring indices for new sample (*T*^2^ and *Q* statistics) with the current thresholds. If both of them are under the thresholds, go to step 4, otherwise, go to step 5.
4. Update the window by including the new sample in the moving window, exclude the oldest ones, and update the PCA model by recalculating it and its thresholds and go to step 1.
5. Report an alarm. Go to step 1.

**Fig 3:**
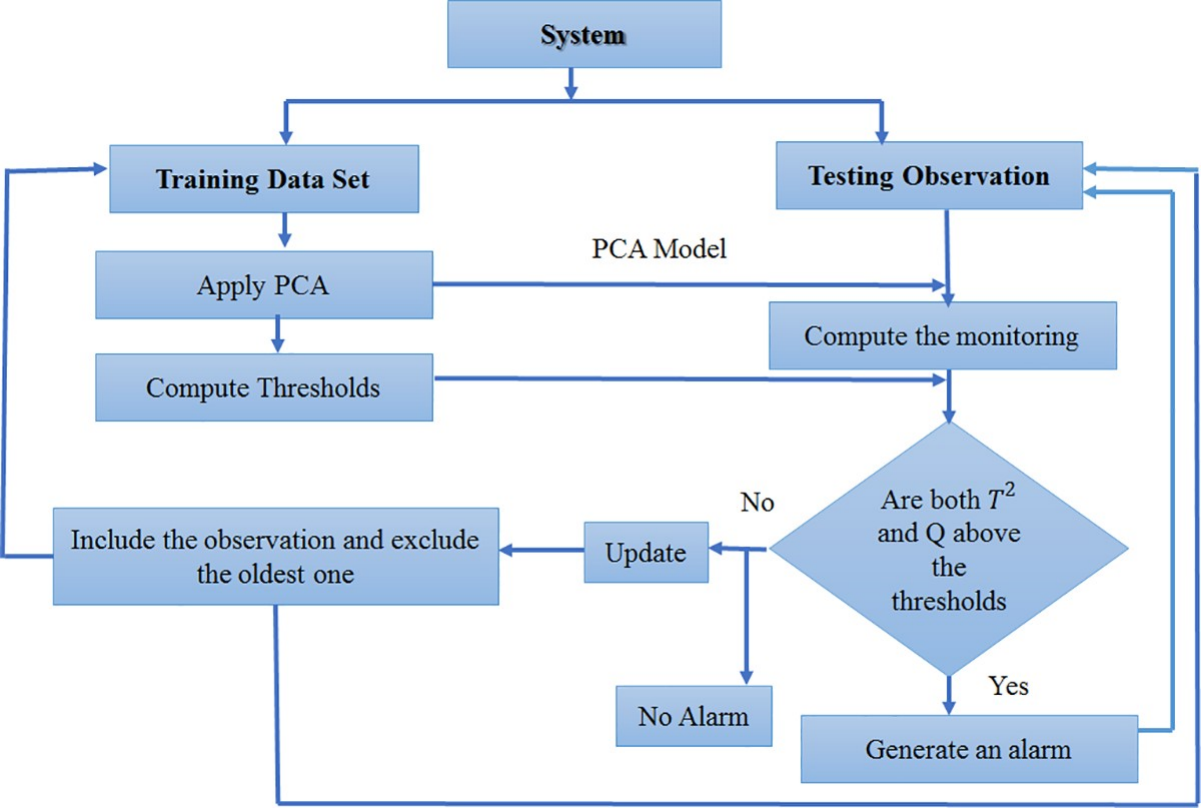
Moving window PCA flowchart [30].

Fig 3 shows the flowchart of the MWPCA model. A detail of equations the MWPCA model can be found in references [15, 17].

## 3. Proposed method (Moving Window Dynamic PCA)

Nowadays, industrial systems are complex, where fast and current observation is highly dependent on past observations. Because of the different state of the system, the collected data are non-stationary. Also, the lags caused by autocorrelation in the variables may not be the same in all variables. Thus, an approach is required that can overcome issues such as dimensionality, non-stationary property, and autocorrelation simultaneously. The proposed method is a data-driven one, which uses the features of two well-known multivariate process monitoring, DPCA and MWPCA. Augmented matrix DPCA technique dominates the autocorrelation property and MWPCA, through the generation of adaptive thresholds, reduces time-varying property. The proposed method, Moving Window Dynamic PCA (MWDPCA) combines the features of methods DPCA and MWPCA to reduce the effects of autocorrelation and time-varying and try to enhance the sensitivity and robustness of process monitoring model. Moving Window Dynamic PCA (MWDPCA) is applied in two offline and online steps. These two phases are illustrated in the form of pseudo-codes as follows:

**Offline phase:**

**For** i = 1: p (p number of variable)

Determine the optimum lag for *i*^*th*^ variable by AIC criterion.

**End for i**

Arrange the augmented matrix of the data by adding lag variables.

Set α such that 1- α is a given confidence level.

Construct PCA form augmented matrix (Mean, Standard Deviation and number of PCs)

Compute the initial thresholds, *Q*′ and *T*^2′^ using Eqs (8) and (10)

Determine the length of the window using training data and based on minimizing FAR and name it H.

Set initiate the binary fault indicator to zero

Set n: number of rows of the training data set

Set Q_off = T2_off = [0]_1×*n*_

**For** i = 1: n

Set x: *i*^*th*^ a row of training data set

Compute x* standardized of x by the mean and standard deviation of the previous step

Compute *Q* and *T*^2^ corresponding x* using Eqs (7) and (9).

Set *i*^*th*^ component of Q_off and T2_off, respectively Q and *T*^2^

**End for i**

Set *Q*_*fixed*_ and $T_{fixed}^{2}$, quantile 1- α percent of Q_off and T2_off

Online phase:

Set m: number of rows of the testing data set

**For** i = 1:m

Set x: *i*^*th*^ a row of testing data set and add it lags

Compute x* standardized of x by the mean and standard deviation of the previous step (in offline phase)

Evaluate the corresponding *Q* and *T*^2^ for x*

**If** (*Q*<*Q*′ *and T*^2^<*T*^2′^)

Include the sample with its lags in the window and exclude the oldest one.

Recalculate DPCA form expanded matrix (Mean, Standard Deviation and number of PCs)

Calculate the adaptive thresholds, *Q*" and *T*^2"^ by Eqs (8) and (10)

Set *Q*′ = *Q*"; *T*^2′^ = *T*^2"^.

Set the binary fault indicator to 0

**Else**

Set the adaptive thresholds: $Q^{”}=Q_{fixed};{T^{2}}^{”}=T_{fixed}^{2}$

Set *Q*′ = *Q*”; *T*^2′^ = *T*^2”^.

Set the binary fault indicator to 1.

**End if**

**End for i.**

The first step in the offline phase is determining the lags of each variable using the training data based on Akaike Information Criterion (AIC) index. Any variable's lag may be different from others. Separately finding the lag for each variable increases model accuracy. We can create an extended training data matrix by adding lags to the data. As Ku et al. proved in DPCA, if enough lags *l* are added in the data matrix, the process monitoring statistics is statistically independent of one moment to another. The next step is to determine the length of the window using training data based on FAR minimization. Then, the mean and standard deviation is calculated and data normalized. Afterward, on the data matrix, the PCA algorithm is implemented and the principal components and the initial value for *Q*′ and *T*^2′^ (Initial threshold) using Eqs (8) and (10) determines. The value 0 is given as the fault indicator. For all observations of the training data by using Eqs (7) and (9), *Q* and *T*^2^ are calculated and selected as fixed thresholds *Q*_*fix*_ and $T_{fix}^{2}$. These thresholds are used when an error is detected, which are set by trial and error in conditions that do not affect the process recovery.

**Fig 4:**
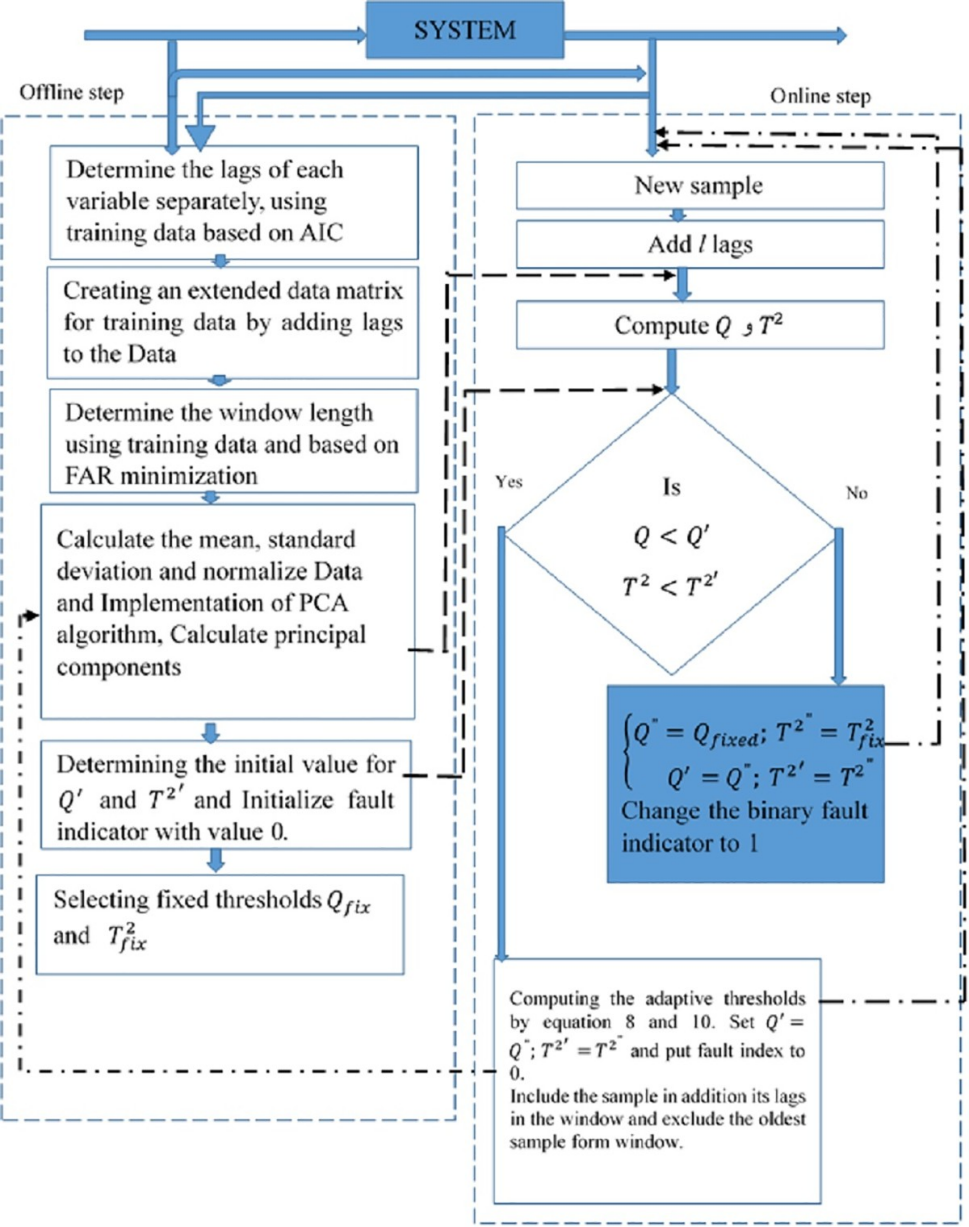
Moving window dynamic PCA flowchart.

The second phase is online. In this step, select a new test sample, expand it by adding *l* lags, and normalize it. Calculate *Q* and *T*^2^ for this sample. Then, *Q* and *T*^2^ are checked with *Q*′ and *T*^2′^ threshold. If both are below the thresholds, this means that the process is under control, and then set the fault index to 0 and include the sample in the window in addition to its lags and exclude the oldest sample form window. The model is updated and computed the adaptive thresholds (*Q*" *and T*^2"^) by Eqs (8) and (10). Set *Q*′ = *Q*"; *T*^2′^ = *T*^2"^. Moving window and constructing adaptive thresholds can cope non-stationary property by limiting the effect of old observations and using close observations to predict the mean and variance and update the model. In other words, the proposed model can detect faults under the different operational modes of the system. But if *Q* and *T*^2^ statistics surpass control limits, set adaptive threshold (*Q*" *and T*^2"^) with *Q*_*fixed*_ and $T_{fixed}^{2}$, and set (*Q*′ = *Q*"; *T*^2′^ = *T*^2"^), then change the binary fault index to 1. Fig 4, illustrates the proposed method (moving window dynamic PCA) in the form of a flowchart.

The proposed method is applied on two applications, including Tennessee Eastman Process and Gas Turbine Exit Temperature Spread.

## 4. Applied MWDPCA method on case study

### 4.1. Eastman Tennessee Process

**Fig 5:**
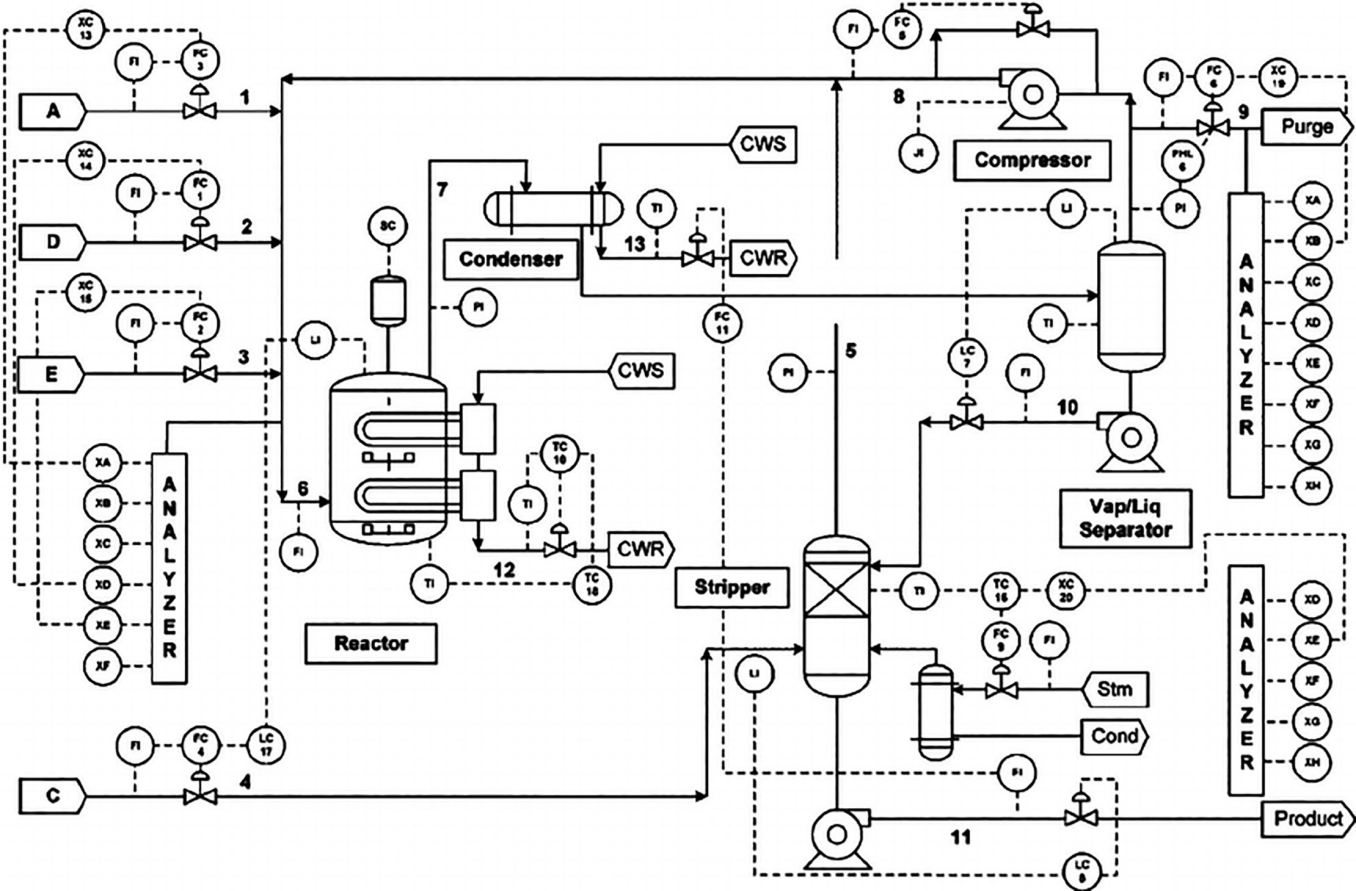
A diagram of the Tennessee Eastman process simulator [13].

The Eastman Tennessee Process (TEP) was developed by Eastman Company, which has created a real industrial process to evaluate the methods developed for process control and monitoring. This simulator is widely used to test and compare various tasks in process control and monitoring. The process has five main performance units: A reactor, a product condenser, a vapor-liquid separator, a recycle compressor, and a product stripper. This process has 41 variables and 12 manipulated variables. Details of these variables are reported in ref [33]. There are 20 faults in this data, as shown in Table 1. Fifteen of them are known, while five of them are unknown. The first seven faults are related to the step change in process variables. Faults 8 to 12 are related to increasing the variability of some process variables. Fault 13 is a slow drift in reactor kinetics, while faults 14 and 15 are associated with sticking valves. Fig 5 reveals the TEP diagram [13].

**Table 1:** Type and description of process faults [33].

Fault number	Description	Type
1	A/C Feed ration, B Composition constant(Stream 4)	Step
2	B Composition, A/C ration constant (Stream4)	Step
3	D Feed temperature (Stream 2)	Step
4	Reactor cooling water inlet temperature	Step
5	Condenser cooling water inlet temperature	Step
6	Feed loss (Stream 1)	Step
7	C Header pressure loss- Reduced availability (Stream 4)	Step
8	A, B, C feed composition (Stream 4)	Random Variation
9	D Feed temperature (Stream 2)	Random Variation
10	C Feed temperature (Stream 4)	Random Variation
11	Reactor cooling water inlet temperature	Random Variation
12	Condenser cooling water inlet temperature	Random Variation
13	Reaction kinetics	Slow drift
14	Reactor cooling water valve	Sticking
15	Condenser cooling water valve	Sticking
16	Unknown	Unknown
17	Unknown	Unknown
18	Unknown	Unknown
19	Unknown	Unknown
20	Unknown	Unknown

**Fig 6:**
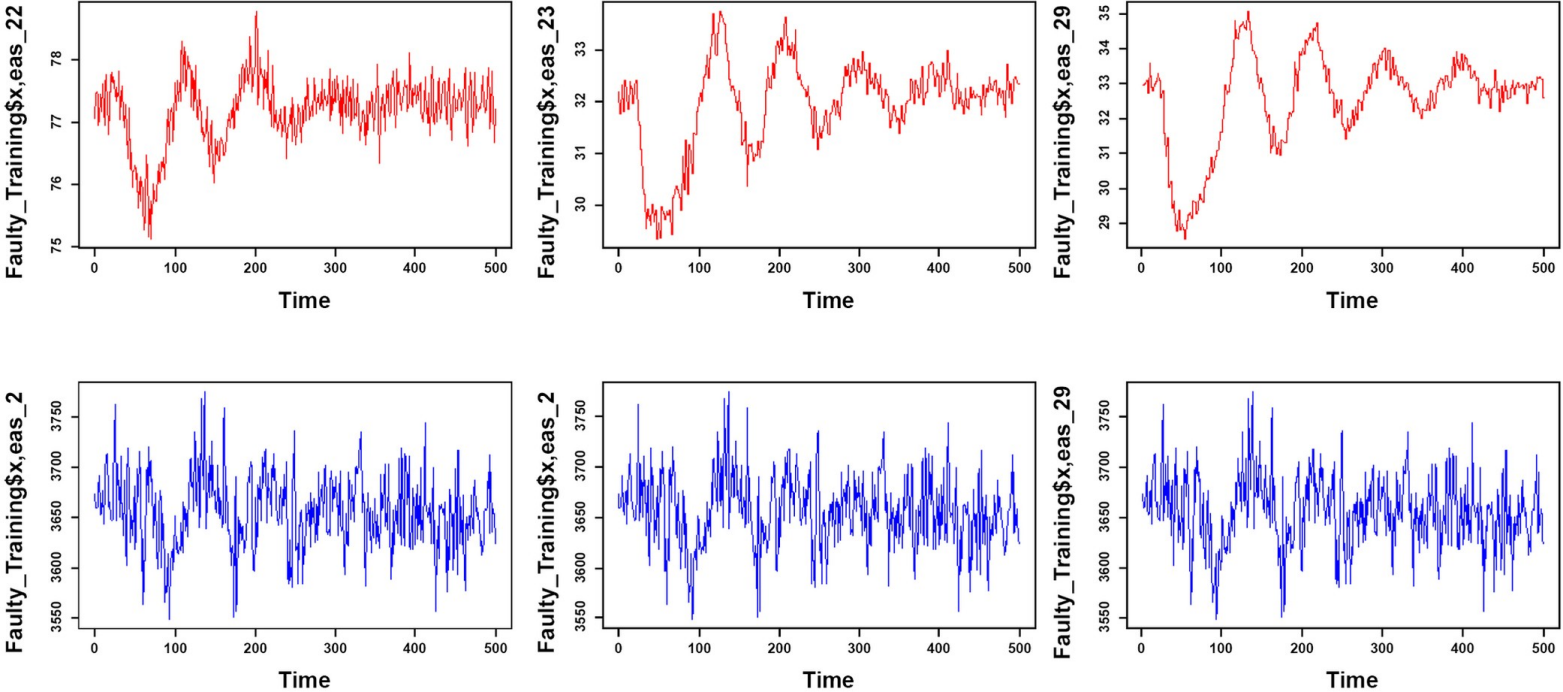
Trends of TEP simulated data for normal process.

The proposed model is implemented on the data employed by Russell et al. [34]. These data have 53 variables which are divided into two sections of training and testing with 500 and 960 observations, respectively. All observations in the training section are under control and there are 20 faults in the observations testing section. The interval between the two observations is three minutes, with each fault reported in the test data after 8 simulation hours of observation. Twenty-one testing datasets were used for this study; the first dataset is fault-free, while the remaining datasets have faults, according to Table 1. Based on the AIC index and KPSS, the simulated data have autocorrelation and non-stationary problems. Fig 6 illustrates a part of this data.

Initially, the number of time lags is calculated for each variable and as mentioned earlier, an expanded matrix is created. Then, the PCA model is implemented on the data, and the number of PCs covering 70% of the total variance is determined. Initial thresholds and adaptive thresholds of the proposed method are calculated at the 99% confidence level. Fixed thresholds are defined experimentally. The window length is selected 40 based on the minimum FAR recorded for the test dataset with no faults. Finally, the proposed method is applied to the observations. A good fault detection technique should have three characteristics: 1- Be robust against the training data set. 2- Be sensitive to all feasible faults in the process. 3- React quickly in fault detection. The robustness is measured by computing the false alarm rate (FAR) upon fault free testing data set. The sensitivity fault detection methods are determined with missed detection rate (MDR) upon faulty testing data set and the promptness fault detection is quantified by calculating delay time detection (DTD) upon faulty testing data set [34]. Performance analysis of the proposed method is evaluated with FAR, DTD, and MDR indices for all the 20 faults of TEP. The results are compared with the results of the approaches introduced by Russell et al. [34], Rato et al. [11], and Sumana et al. [35]. FAR, MDR and DTD indices are calculated by the following equation with the results shown in Tables 2, 3 and 4.

$$FAR=\frac{Number\;of\;normal\;samples\;above\;the\;limits}{Total\;number\;of\;normal\;samples}*100$$ (12)

$$MDR=\frac{Number\;of\;faulty\;samples\;under\;the\;limits}{Total\;number\;of\;faulty\;samples}*100$$ (13)

DTD=faultdetectiontime−faultaccourancetime (14)

**Table 2:** Comparison of FAR for MWDPCA with other methods.

	^*a*^ *PCA*[34]		^*b*^ *DPCA*[34]		^*c*^ *KPCA*[35]		^*d*^ *DKPCA*[35]		*MWDPCA*	
***Statistic***	*Q*	T^2^	*Q*	T^2^	*Q*	T^2^	*Q*	T^2^	*Q*	T^2^
***Training dataset***	*0*.*4*	*0*.*2*	*0*.*4*	*0*.*2*	*1*	*1*	*1*	*1*	*0*	*0*
***Testing dataset***	*1*.*6*	*1*.*4*	*28*.*1*	*0*.*6*	*3*.*65*	*2*.*71*	*2*.*61*	*3*.*02*	*0*	*0*

**Table 3:** Comparison of MDR for MWDPCA with other methods.

*method*	*PCA*[34]		*DPCA*[34]		*MWPCA*[11]		*RPCA*[11]		*KPCA*[35]		*DKPCA*[35]		*MWDPCA*	
*Fault NO*	*Q*	T^2^	*Q*	T^2^	*Q*	T^2^	*Q*	T^2^	*Q*	T^2^	*Q*	T^2^	*Q*	T^2^
***1***	*0*.*3*	*0*.*8*	*0*.*6*	*0*.*5*	*0*.*3*	*0*.*6*	*0*.*9*	*0*.*9*	*0*.*62*	*0*.*99*	.*54*	.*62*	*0*.*49*	*0*.*62*
***2***	*1*.*4*	*2*	*1*.*5*	*1*.*9*	*3*.*1*	*1*.*9*	*3*.*2*	*1*.*7*	*1*.*87*	*2*.*86*	*1*.*72*	*1*.*62*	***1*.*4***	***1*.*60***
***3***	*99*.*1*	*99*.*8*	*99*	*99*.*1*	*99*.*6*	*99*.*4*	*99*.*8*	*99*.*9*	*89*.*7*	*96*.*2*	*94*.*5*	*93*.*3*	*98*.*2*	*98*.*75*
***4***	*3*.*8*	*95*.*6*	*0*	*93*.*9*	*99*.*4*	*99*.*4*	*8*.*90*	*85*.*6*	*39*.*6*	*92*.*3*	*44*.*9*	*81*.*9*	*0*.*1*	***52***
***5***	*74*.*6*	*77*.*5*	*74*.*8*	*75*.*8*	*78*.*5*	*76*.*7*	*90*.*1*	*77*.*9*	*68*.*7*	*71*.*7*	*71*.*9*	*70*.*5*	***50*.*6***	***64***
***6***	*0*	*1*.*1*	*0*	*1*.*3*	*0*.*1*	*0*.*5*	*0*.*1*	*0*.*7*	*0*.*62*	*0*.*75*	*0*.*5*	*0*.*62*	*0*.*12*	*0*.*5*
***7***	*0*	*8*.*5*	*0*	*15*.*9*	*6*	*0*.*1*	*30*	*0*.*1*	*0*	*54*.*6*	*0*	*0*.*24*	*0*.*1*	*0*.*1*
***8***	*2*.*4*	*3*.*4*	*2*.*5*	*2*.*8*	*3*.*2*	*2*.*9*	*15*.*6*	*2*.*9*	*2*.*62*	*12*	*2*.*5*	*2*.*74*	***2*.*3***	***2*.*62***
***9***	*98*.*1*	*99*.*4*	*99*.*4*	*99*.*5*	*99*.*9*	*99*.*9*	*100*	*99*.*8*	*89*.*8*	*96*.*7*	*93*.*6*	*93*.*2*	*91*.*7*	***90***
***10***	*65*.*9*	*66*.*6*	*66*.*5*	*58*	*98*.*9*	*98*.*4*	*99*.*6*	*99*.*6*	*43*.*2*	*59*.*6*	*45*.*3*	*45*	***21***	***31*.*2***
***11***	*35*.*6*	*79*.*4*	*19*.*3*	*80*.*1*	*98*.*4*	*98*.*1*	*93*.*5*	*92*.*3*	*41*.*3*	*90*.*9*	*41*.*6*	*61*.*5*	***4*.*2***	***34*.*7***
***12***	*2*.*5*	*2*.*9*	*2*.*4*	*1*	*2*.*7*	*1*.*7*	*13*.*5*	*2*.*0*	*1*.*36*	*10*.*3*	*1*.*38*	*1*.*38*	***0*.*3***	***0*.*3***
***13***	*4*.*5*	*6*	*4*.*9*	*4*.*9*	*0*.*2*	*2*.*4*	*6*	*5*.*7*	*5*.*62*	*7*.*12*	*5*.*62*	*5*.*62*	*4*.*88*	*5*
***14***	*0*	*15*.*8*	*0*	*6*.*1*	*0*.*1*	*0*.*1*	*12*.*6*	*1*.*9*	*0*	*61*.*1*	*0*	*0*	*0*.*12*	*0*.*37*
***15***	*97*.*3*	*98*.*8*	*97*.*6*	*96*.*4*	*99*.*8*	*99*.*0*	*99*.*8*	*100*	*82*.*1*	*91*.*1*	*85*.*4*	*83*.*7*	*92*.*9*	*93*.*9*
***16***	*75*.*5*	*83*.*4*	*70*.*8*	*87*.*3*	*99*.*6*	*99*.*9*	*99*.*5*	*100*	*58*.*6*	*74*.*1*	*60*.*9*	*59*.*9*	***40*.*6***	***53*.*2***
***17***	*10*.*8*	*25*.*9*	*5*.*3*	*24*	*78*.*2*	*80*.*3*	*10*.*9*	*24*.*6*	*12*.*2*	*38*.*4*	*13*.*1*	*17*.*6*	***2*.*45***	***7*.*1***
***18***	*10*.*1*	*11*.*3*	*10*	*11*.*1*	*11*.*2*	*11*.*0*	*10*.*5*	*10*.*9*	*10*.*8*	*11*.*8*	*10*.*7*	*11*	***8*.*07***	***8*.*57***
***19***	*87*.*3*	*99*.*6*	*73*.*5*	*99*.*3*	*99*.*5*	*99*.*3*	*100*	*99*.*6*	*95*.*2*	*98*.*1*	*95*	*98*	***28*.*9***	***78*.*7***
***20***	*55*	*70*.*1*	*49*	*64*.*4*	*94*	*92*.*6*	*90*.*6*	*89*.*8*	*42*.*6*	*68*.*8*	*45*.*4*	*48*.*3*	***14*.*6***	***28*.*7***
***MMDR***	*36*.*2*	*47*.*4*	*33*.*8*	*46*.*1*	*53*.*6*	*53*.*2*	*49*.*2*	*49*.*8*	*34*.*3*	*51*.*9*	*35*.*7*	*38*.*8*	***23*.*1***	***32*.*6***

**Table 4:** Comparison of DTD for MWDPCA with other methods (sample).

*method*	*PCA*[34]		*DPCA*[34]		*KPCA*[35]		*DKPCA*[35]		*MWDPCA*[35]	
*Fault NO*	*Q*	T^2^	*Q*	T^2^	*Q*	T^2^	*Q*	T^2^	*Q*	T^2^
***1***	*3*	*7*	*5*	*6*	*6*	*8*	*5*	*6*	***4***	***5***
***2***	*12*	*17*	*13*	*16*	*16*	*24*	*15*	*14*	***11***	***12***
***3***	*-*	*-*	*-*	*-*	*84*	*358*	*84*	*84*	*215*	*664*
***4***	*3*	*-*	*1*	*151*	*8*	*60*	*8*	*58*	***1***	***3***
***5***	*1*	*16*	*2*	*2*	*12*	*15*	*1*	*12*	*2*	***2***
***6***	*1*	*10*	*1*	*11*	*6*	*7*	*5*	*6*	***1***	***4***
***7***	*1*	*1*	*1*	*1*	*1*	*3*	*0*	*0*	***1***	***1***
***8***	*20*	*23*	*21*	*23*	*22*	*11*	*21*	*23*	***19***	*21*
***9***	*-*	*-*	*-*	*-*	*6*	*320*	*1*	*4*	*74*	*664*
***10***	*49*	*96*	*50*	*101*	*20*	*24*	*23*	*16*	*26*	*26*
***11***	*11*	*304*	*7*	*195*	*52*	*296*	*48*	*141*	***7***	***9***
***12***	*8*	*22*	*8*	*3*	*8*	*24*	*7*	*7*	***3***	***3***
***13***	*37*	*49*	*40*	*45*	*46*	*51*	*46*	*46*	*38*	***38***
***14***	*1*	*4*	*1*	*6*	*1*	*45*	*1*	*1*	*1*	*3*
***15***	*740*	*-*	*-*	*-*	*576*	*616*	*576*	*576*	***215***	***242***
***16***	*197*	*312*	*196*	*199*	*197*	*225*	*35*	*192*	***22***	***26***
***17***	*25*	*29*	*24*	*28*	*27*	*32*	*27*	*28*	***23***	***28***
***18***	*84*	*93*	*84*	*93*	*88*	*96*	*87*	*89*	***57***	***69***
***19***	*-*	*-*	*82*	*-*	*100*	*100*	*100*	*100*	***9***	***19***
***20***	*87*	*87*	*84*	*89*	*80*	*82*	*80*	*82*	***48***	***55***

The numbers written in the table indicate the number of observations from the moment the fault occurred to the moment it was detected. An empty cell means that DTD has not been reported.

**Fig 7:**
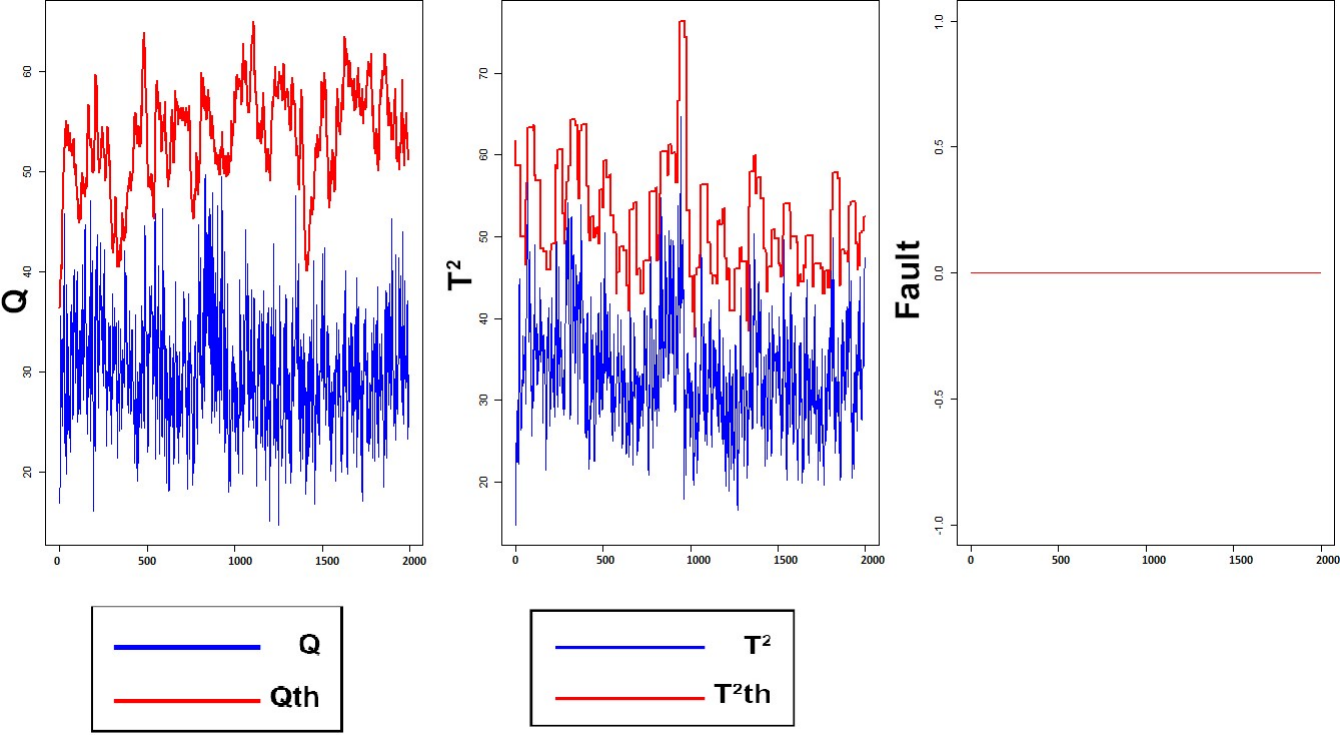
Monitoring performance of MWDPCA for normal process.

According to the results of Table 2 and Fig 7, the proposed method, in both training and test sets, has been successfully minimized FAR index for both *Q* and *T*^2^ charts. These results indicate that the proposed method, compared to other methods listed in Table 2 has high potential terms of reducing FAR and it is robust independent of the training data set. It should be noted that Rato et al. [11] did not provide a report on the FAR index for implementation MWPCA and RPCA methods on TEP data.

Table 3 shows the results of MDR for PCA, DPCA, MWPCA, RPCA, KPCA, KDPCA and proposed method. A comparison of the results shows that the proposed method has been able to significantly reduce the MDR index in some faults simultaneously in both *T*^2^ and Q or in *T*^2^ or Q. To evaluate the overall performance of the proposed method in the MDR (MMDR) index, the mean MDR index is calculated. The results show that the proposed method has a better overall performance in both *T*^2^ and Q charts compared to other methods, and it is more sensitive to possible faults.

Table 4 presents the Detection Time Delay in the proposed method and compares it with the DTD index in PCA, DPCA, Kernel PCA, and Kernel Dynamic PCA methods. The numbers written in the table indicate the number of observations from the moment the fault occurred to the moment it was detected.

According to the results in Table 4, the bold numbers indicate a better performance of the proposed method in DTD index for *Q* or *T*^2^ charts or both simultaneously as compared to other methods. As shown in Table 4, the proposed method has a more agile performance about fault 4, 5, 6, 7, 11, 12 and 14. An empty cell means that DTD has not been reported. Moreover, Rato et al. [11] did not provide a report on the DTD index in their research.

**Fig 8:**
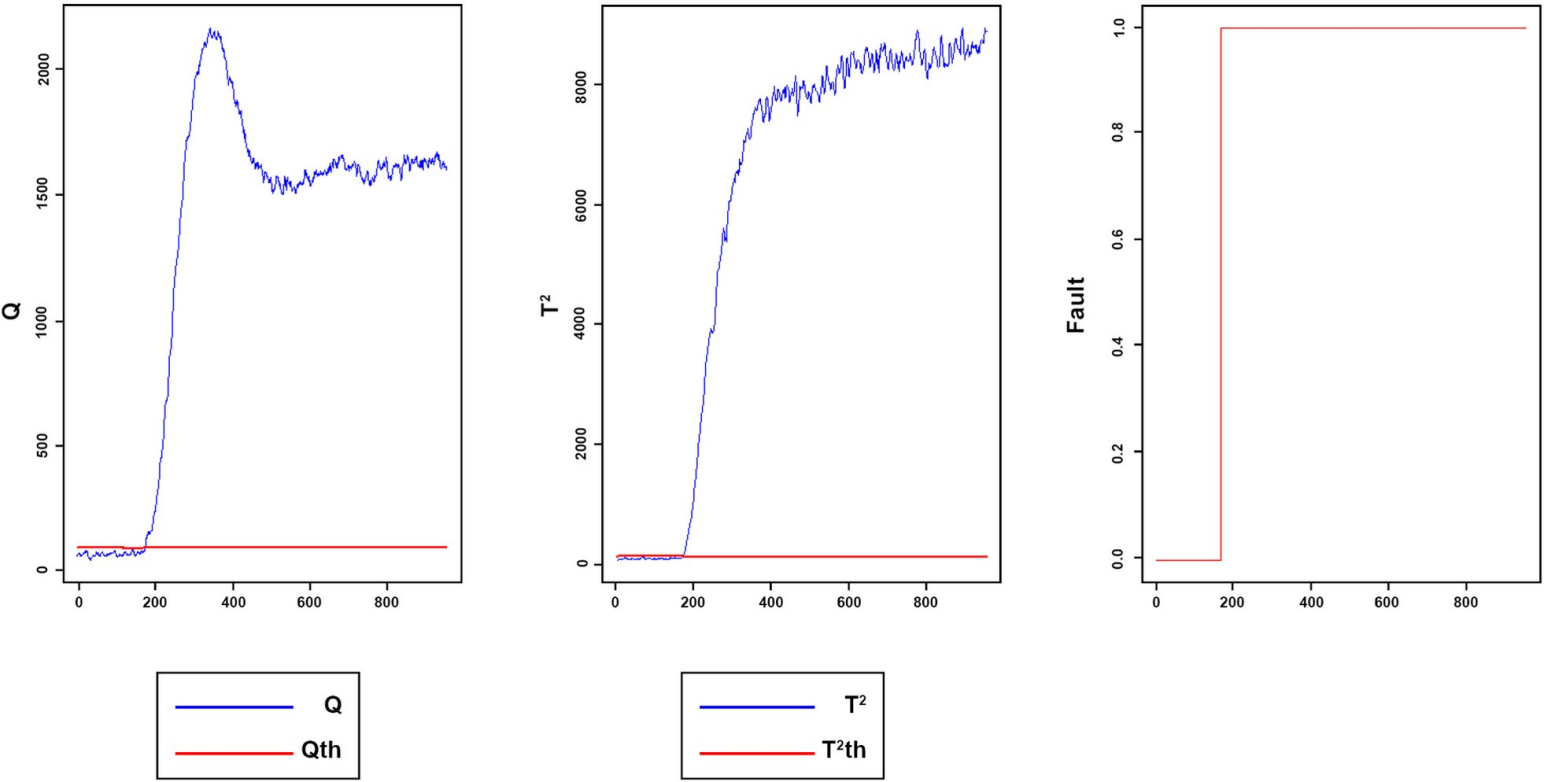
Monitoring performance of MWDPCA for fault 2.

**Fig 9:**
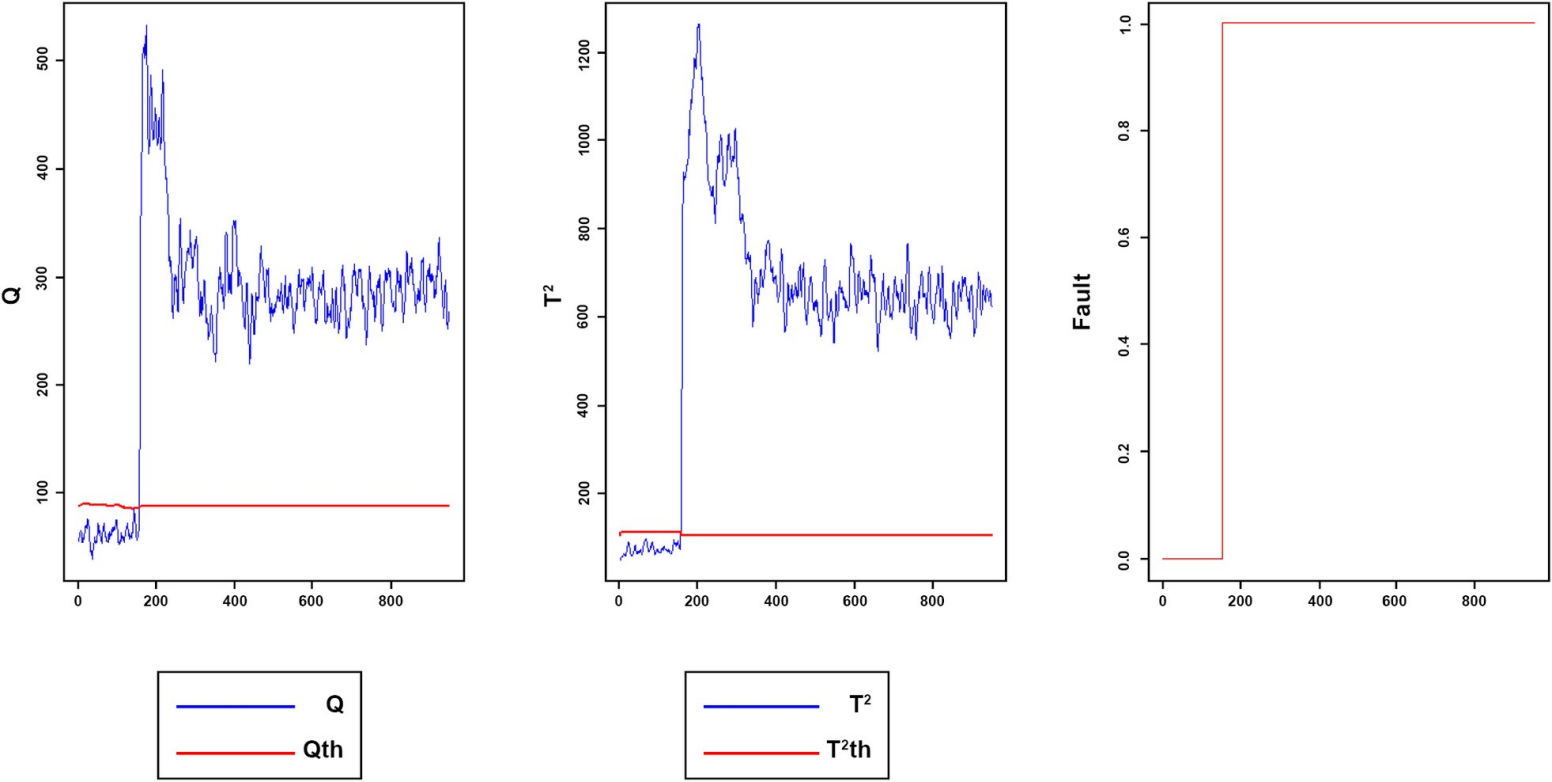
Monitoring performance of MWDPCA for fault 7.

**Fig 10:**
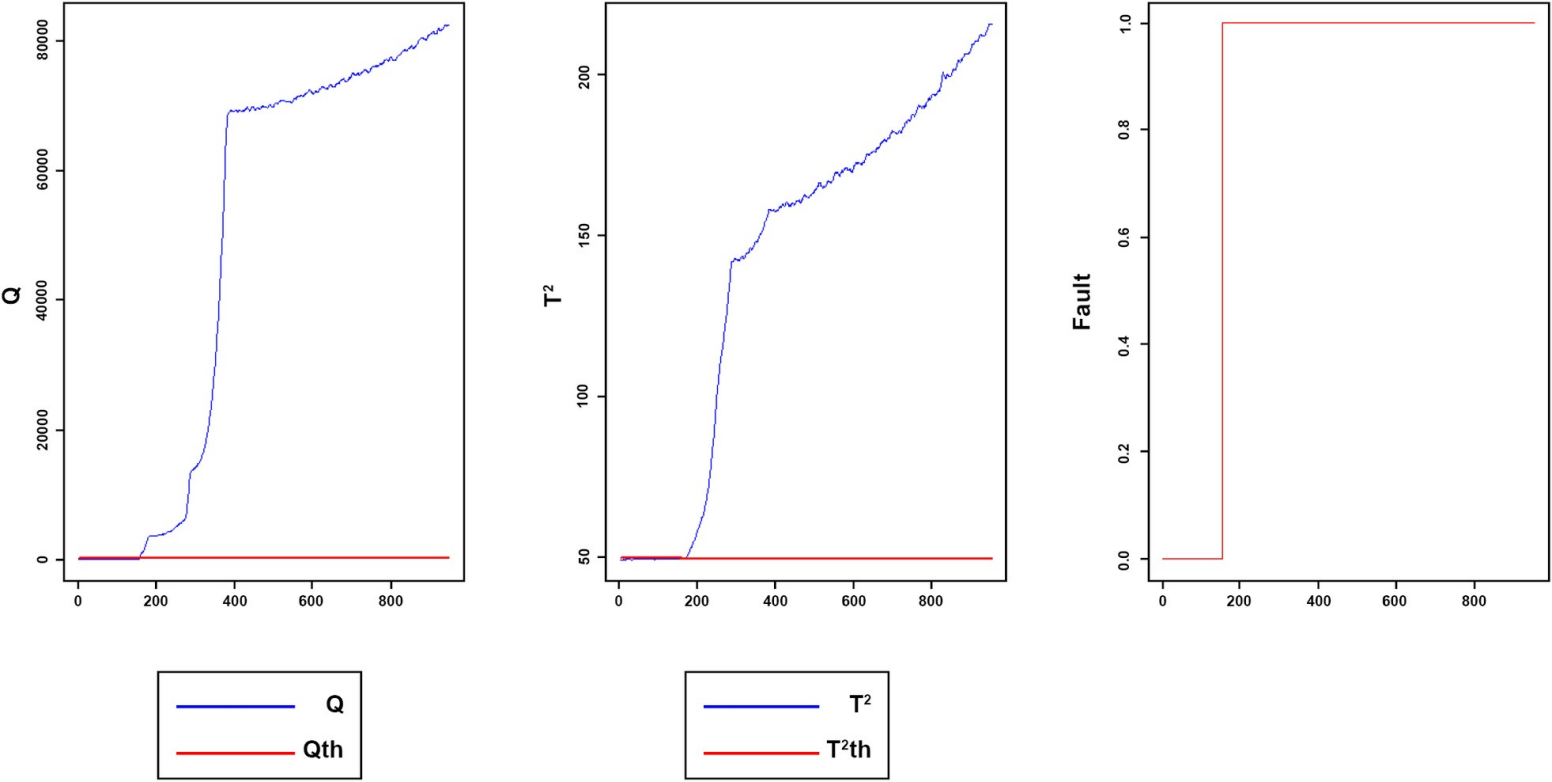
Monitoring performance of MWDPCA for fault 6.

**Fig 11:**
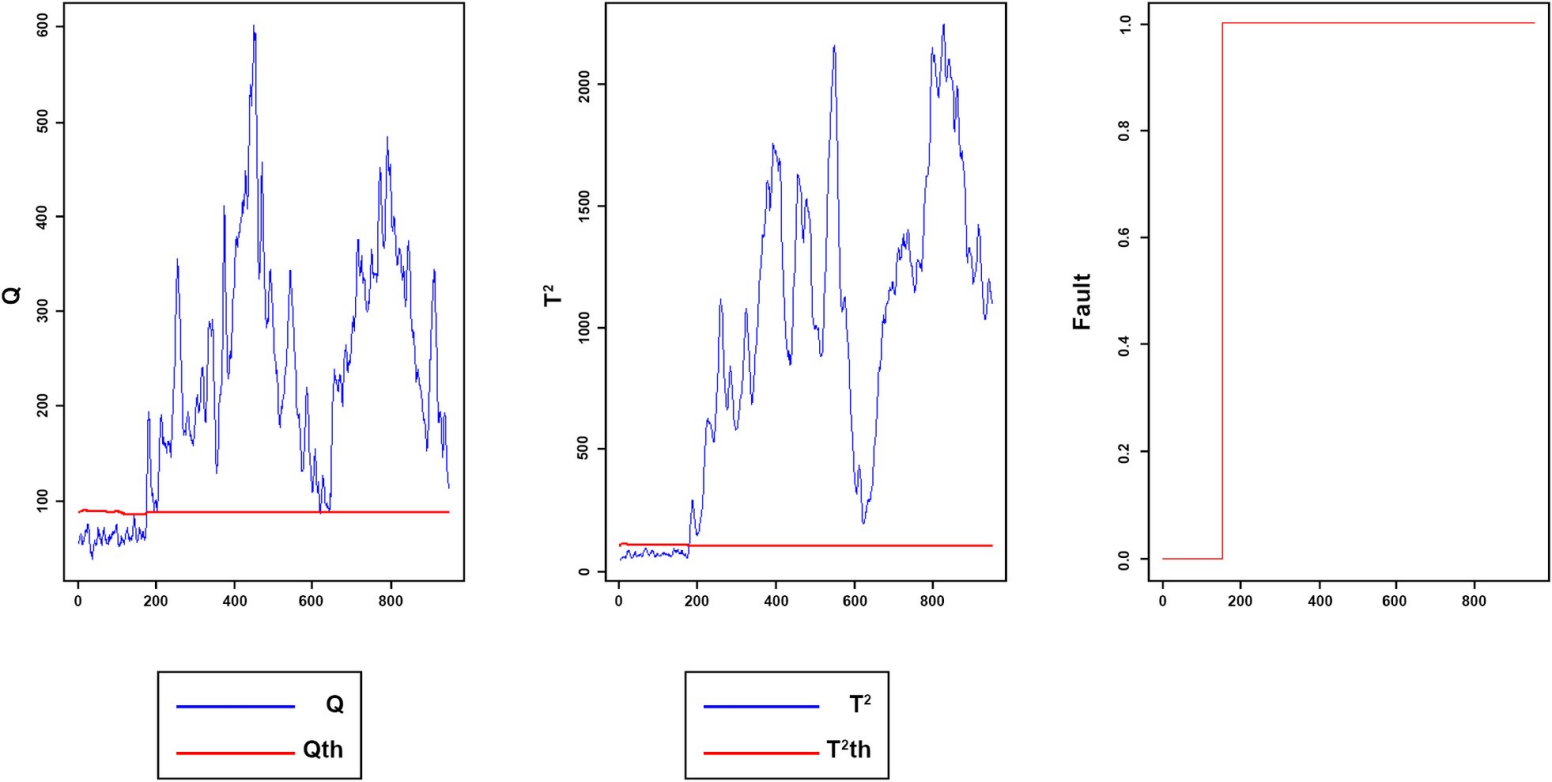
Monitoring performance of MWDPCA for fault 8.

**Fig 12:**
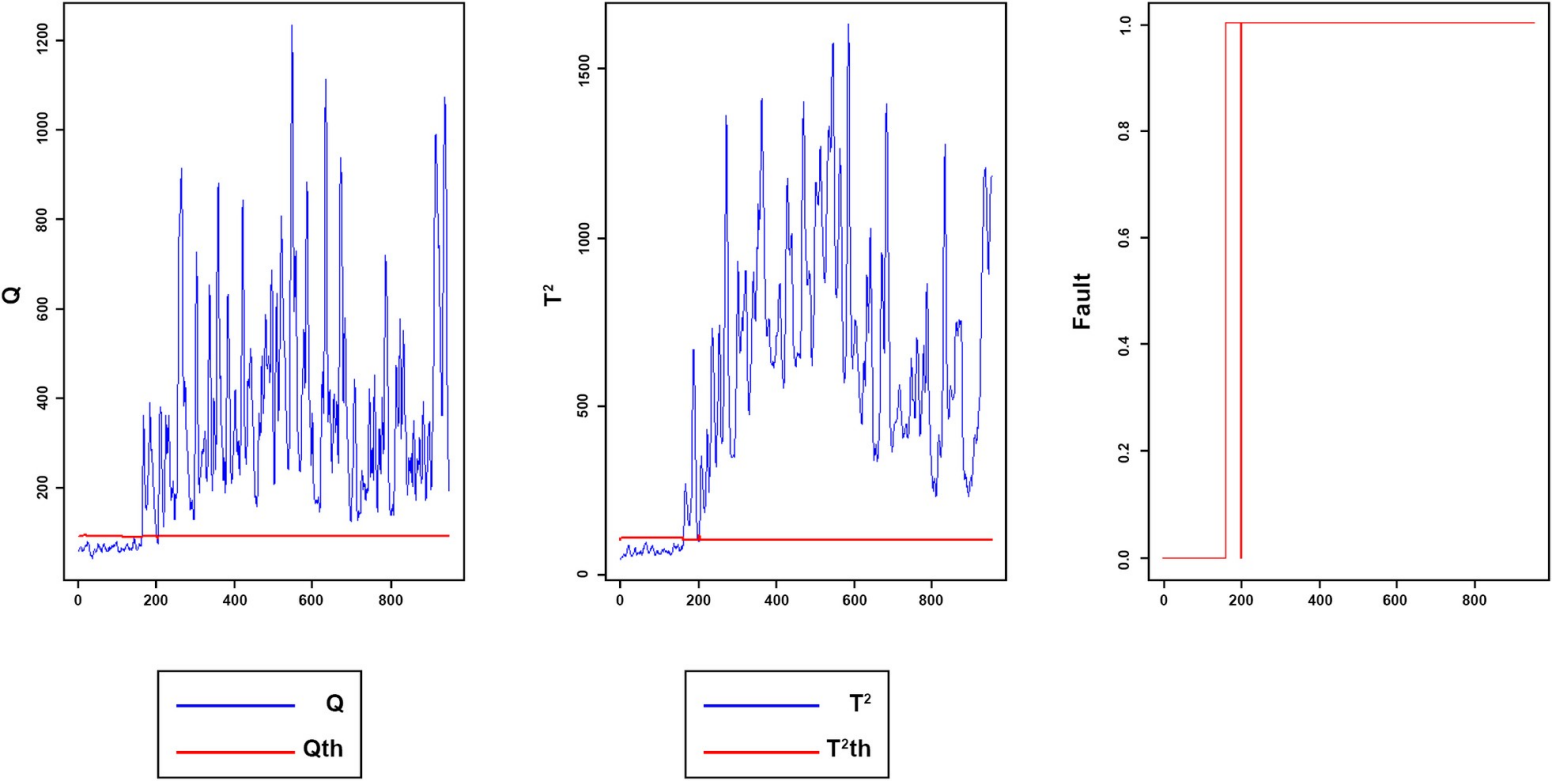
Monitoring performance of MWDPCA for fault 12.

**Fig 13:**
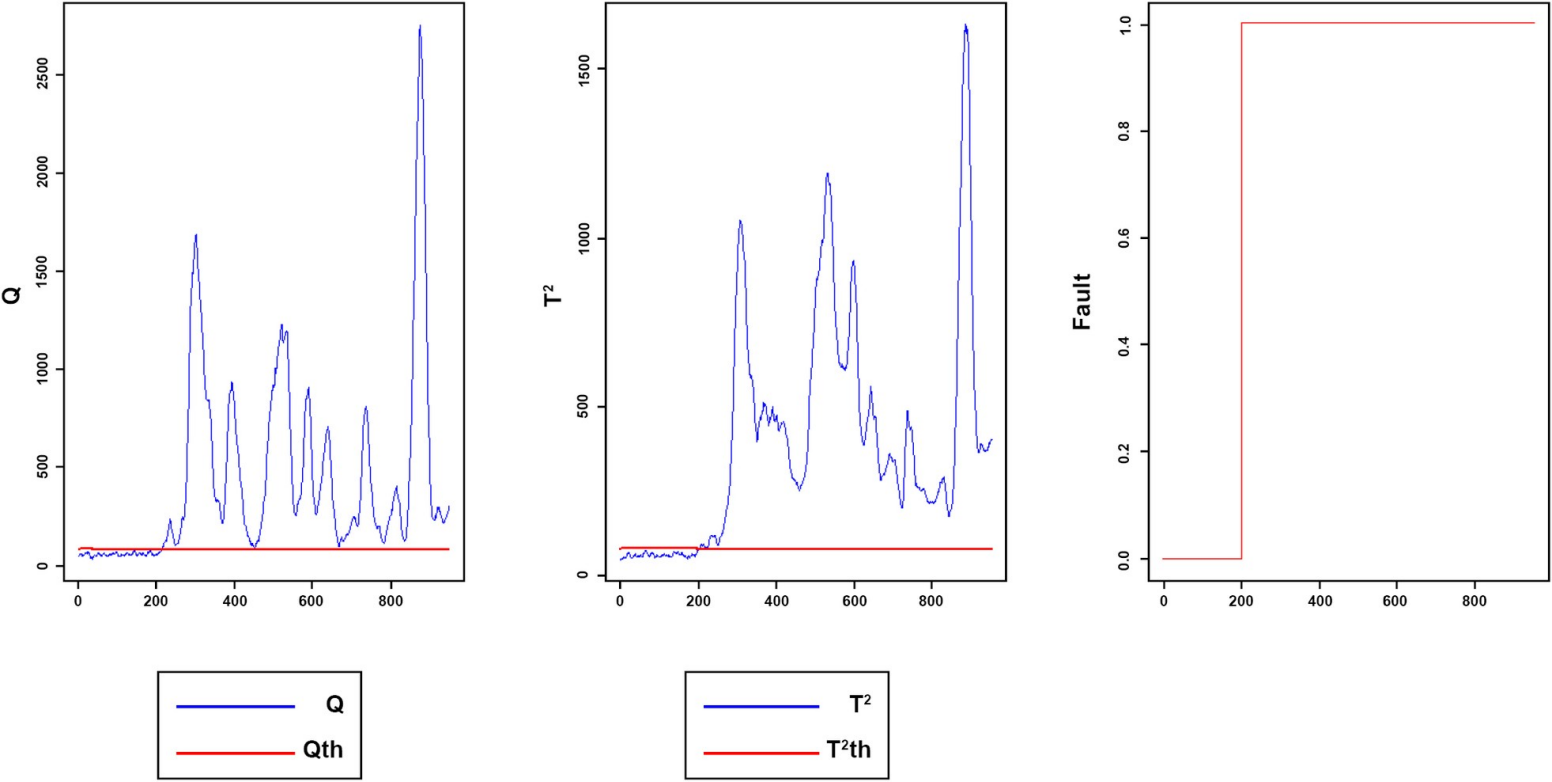
Monitoring performance of MWDPCA for fault 13.

**Fig 14:**
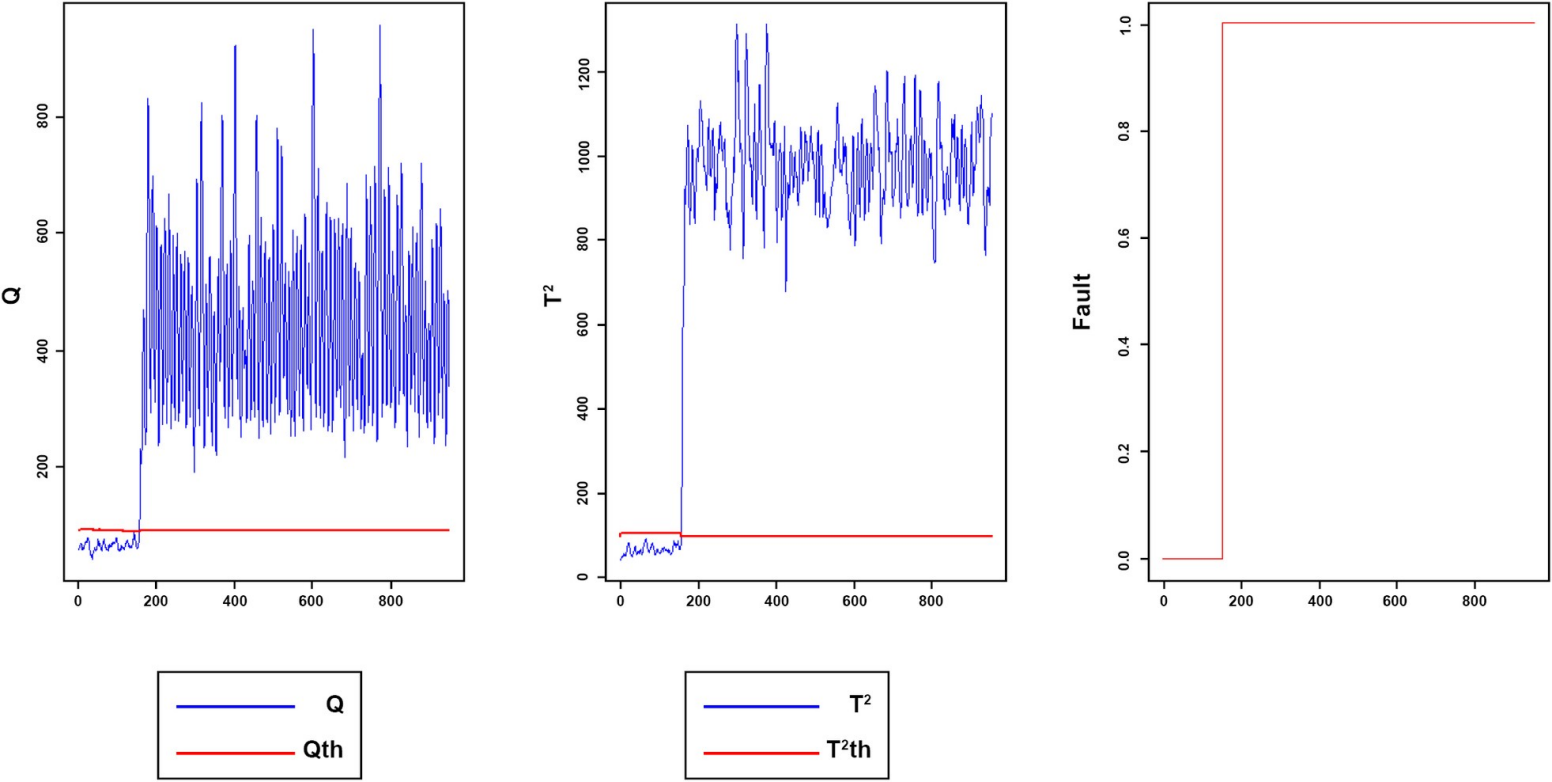
Monitoring performance of MWDPCA for fault 14.

**Fig 15:**
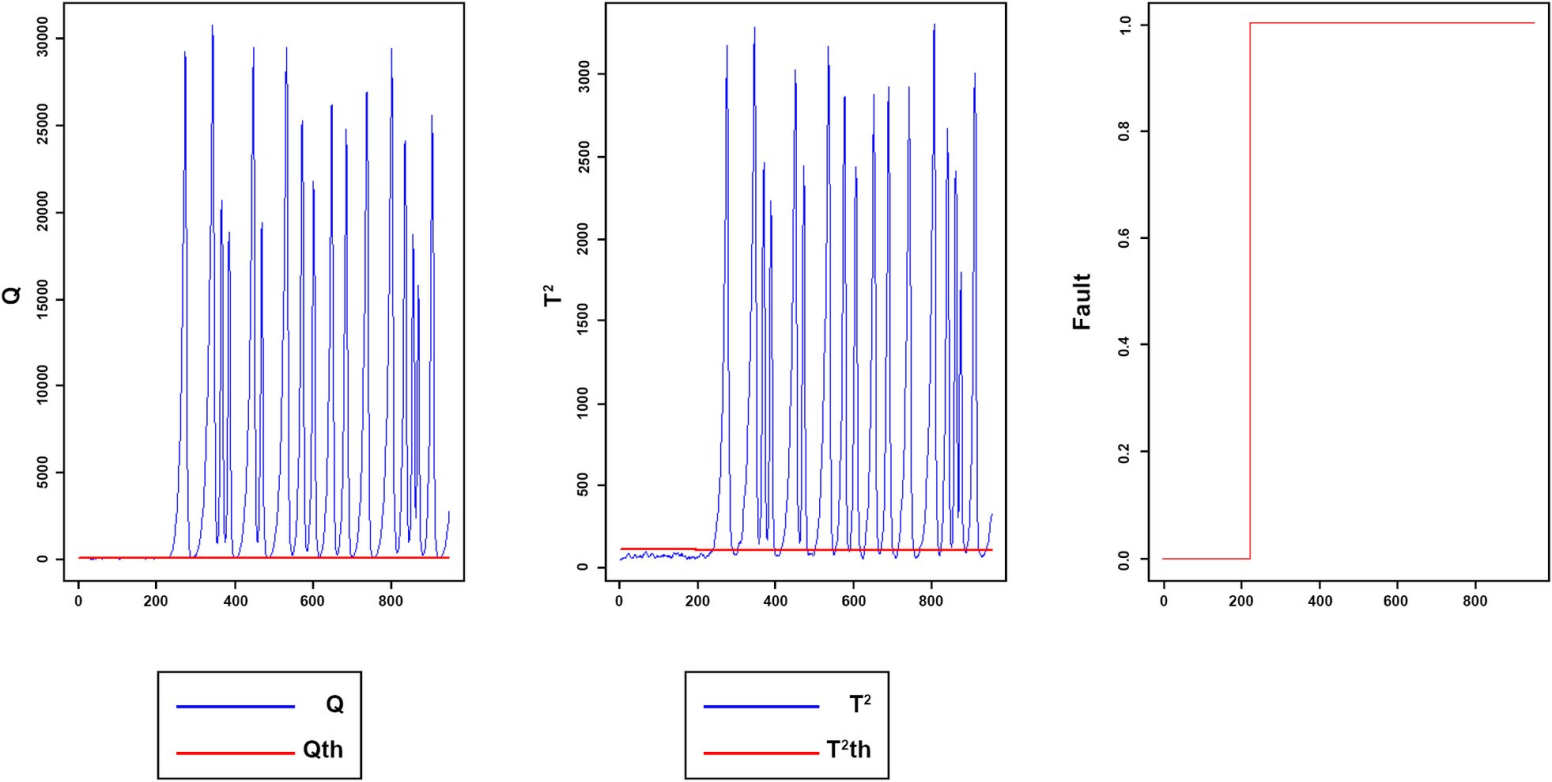
Monitoring performance of MWDPCA for fault 17.

**Fig 16:**
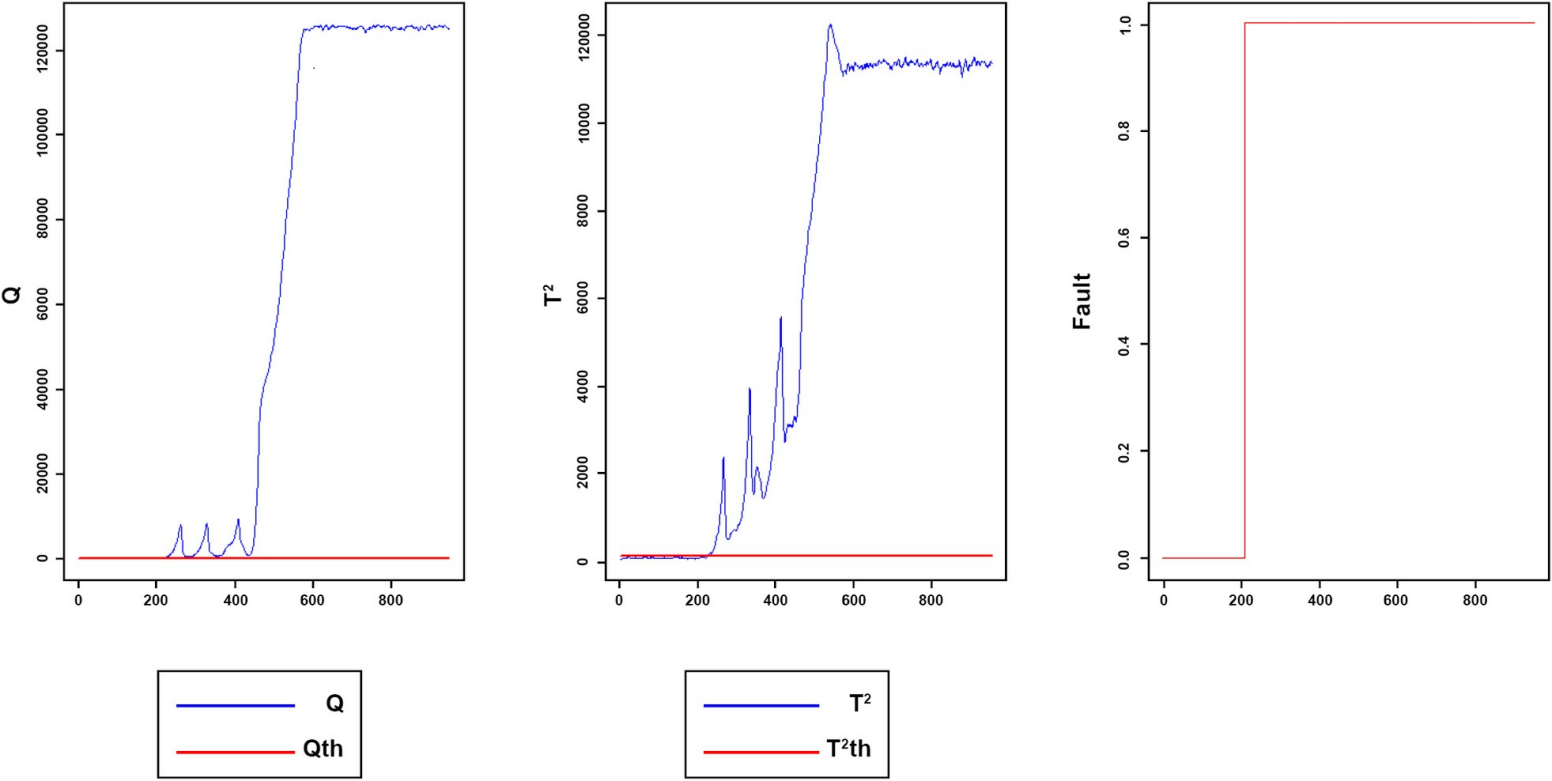
Monitoring performance of MWDPCA for fault 18.

Figs 8–16 present the Q and *T*^2^ control charts drawn for the proposed method with their adaptive thresholds. The first chart from the left is *Q* control chart, the second and third charts indicate monitoring by *T*^2^ and fault indicator chart, respectively. In the *Q* and *T*^2^ control charts, the red lines represent the *Q*_α_ and $T_{\alpha }^{2}$ adaptive thresholds and the blue dots represent the calculated values of *Q* and *T*^2^ for each sample. As long as the blue dots in both charts are below the threshold, the process is under control. The third chart illustrates the fault indicator, which is a binary variable. In this chart, a value 1 means that the process is under control, and a value 0 indicates that the sample has exceeded the threshold. For example, fault 7 (Fig 9) indicates that the process was under control until sample 159. The fault occurred in sample 160 afterward and the method has detected fault in sample 161. Figs 8 and 10 illustrate the detection of step-change fault. Detecting faults number 8 and 12 regarding random Variation is shown in Figs 11 and 12. Figs 13 and 14 represent slow drift and Sticking faults, respectively. Detecting unknown faults are shown in Figs 15 and 16.

### 4.2. MWDPCA applied to turbine exhaust temperature spread

Gas turbines are designed for many different purposes. In the industry, they are commonly used to drive compressors to transport gas through pipelines and generators that produce electrical power [36]. In the past, GTs use was generally limited to generating electricity in periods of peak electricity demand; however, nowadays, they are being used in combined cycle power plants for base load production [37]. Consequently, their availability, as well as reliability, play a significant role in these machines. The development of a gas turbine in recent years has been facilitated most considerably by three factors:

- Metallurgical developments that can be used to apply high temperatures in the combustor and turbine components;
- The increased underlying knowledge of aerodynamics and thermodynamics;
- Designing and simulating turbine airfoils as well as combustor and turbine blade cooling configurations by computer software.

After compressing the air in the compressor, the fuel is injected into it and combustion increases the temperature of the gas. Turbine inlet temperature (TIT) is defined as the average temperature of the flue gas that faces the first stage of turbine blades. During the expansion of the flue gas in the turbine, the pressure and temperature drop and the flue gas leaving the turbine with turbine exhaust temperature (TET).

The efficiency and specific power of GT would be improved if TIT could be increased. Nevertheless, designing and manufacturing turbines resisting higher TIT is a technological limit. Since TIT is too hot to be measured directly, it is usually calculated by measuring TET. Due to the rotation and turbulence of the flue gas stream, both TET and TIT have a profile on their section. In v94.2 GTs, TET is measured by six temperature transmitters to indicate a precise profile. GT manufacturers use various methods to calculate the TIT regarding the measured TET; by monitoring the TET, the operator tries to keep the GT under protected conditions.

**Fig 17:**
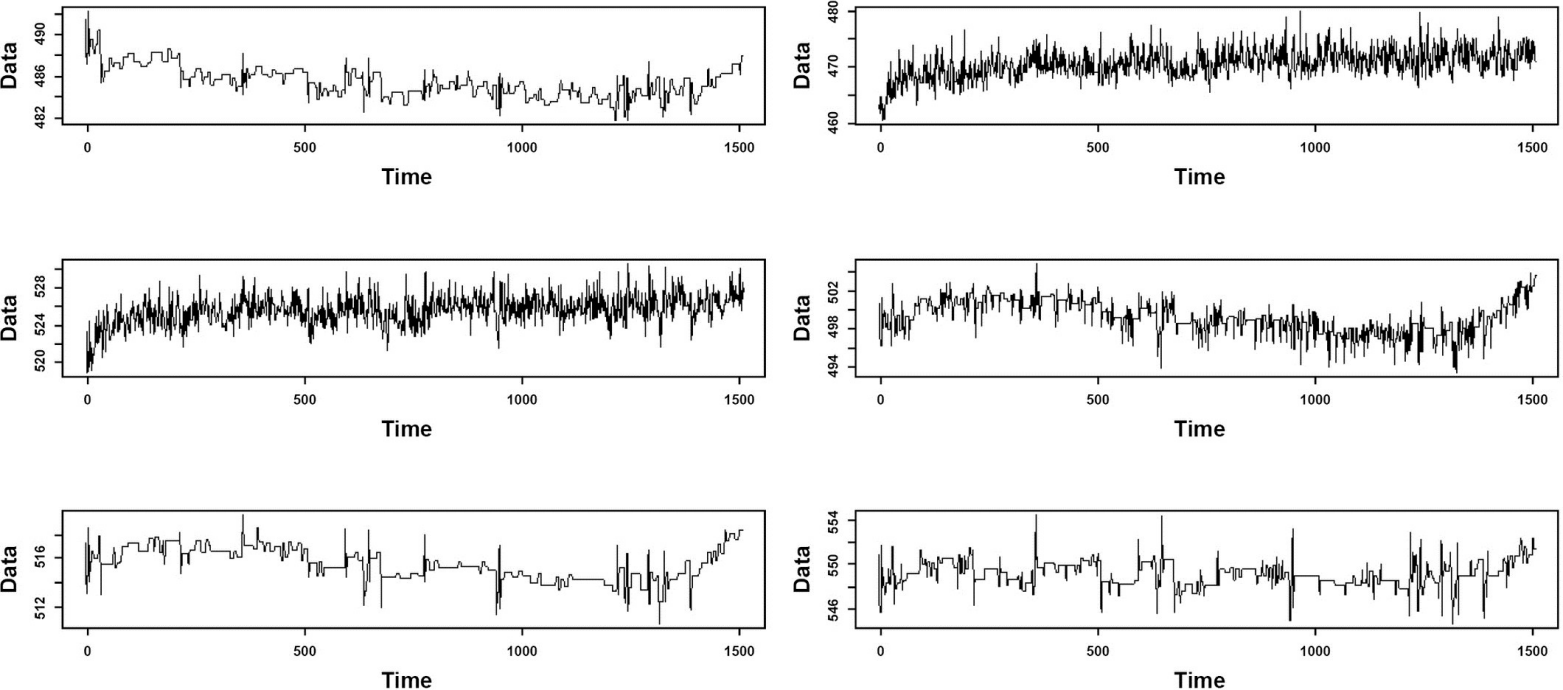
Trends of TET data for normal process.

Throughout this paper, TET of an Iranian gas turbine company was used to illustrate the behavior of the proposed method. As observed in Fig 17, the data consist of measurements of six sensors, with each sensor representing TET of V94-2 gas turbine measured approximately in a 1500-minute time period. The samples were recorded every 1 minute. Statistical tests were implemented to detect the behavior of the data. These data are non-stationary in accordance with the test of KPSS and auto-correlated with AIC index. In this subsection, MWDPAC model was applied on TET data for controlling the behavior of GT and early fault detection.

Preprocessing is normally done in various fields. The type of preprocessing depends on the type of the process. In the case of the TET data, no special preprocessing is necessary; standardizing the data was the only necessary procedure. The first 200 observations were used to training data set. Note that these data were collected when all parameters were under control. There are two kinds of fault in testing data: i) step change which means occurrence of sudden faults; ii) slow ramp.

**Fig 18:**
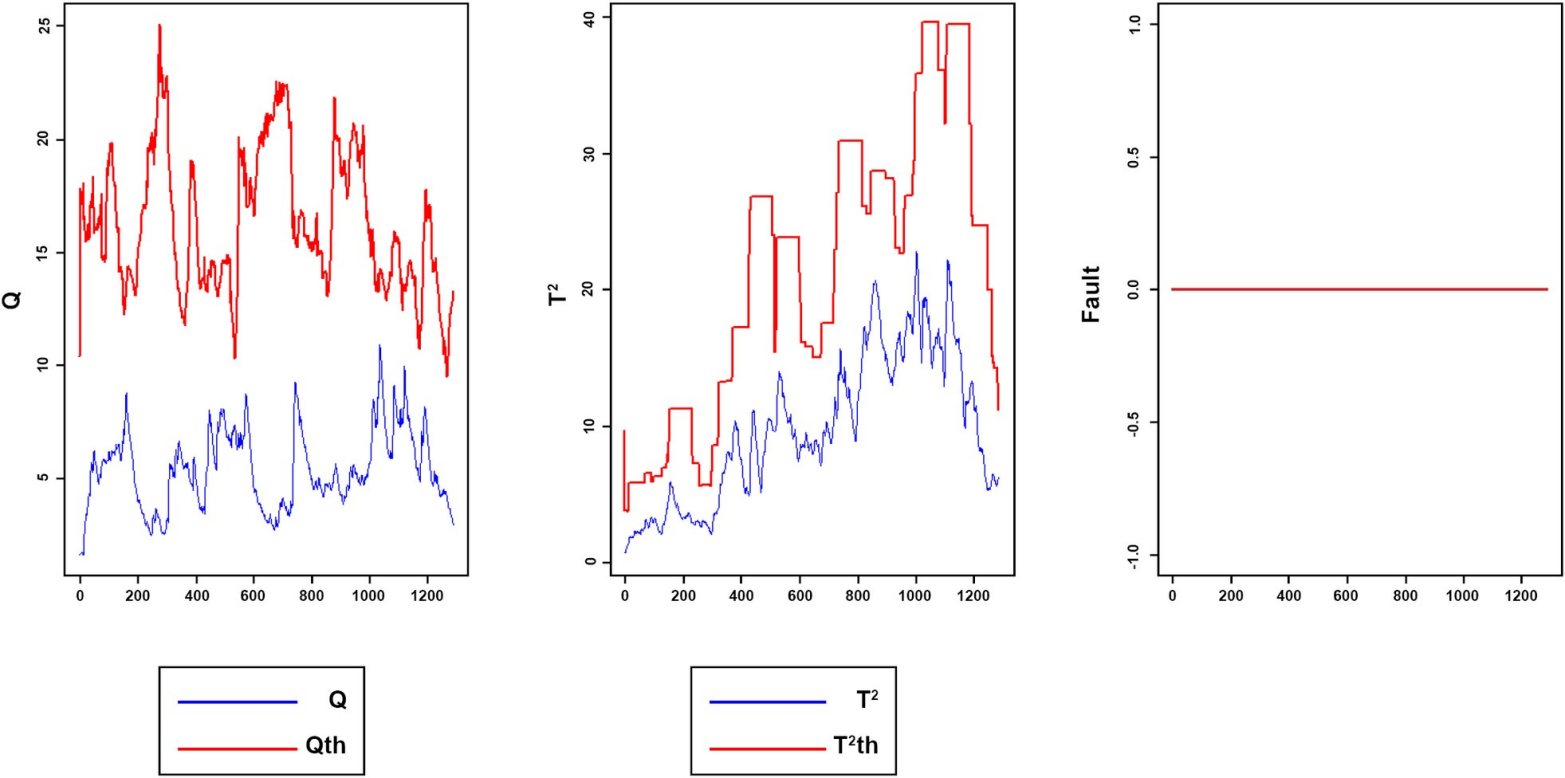
Monitoring performance of MWDPCA for TET data normal process.

**Fig 19:**
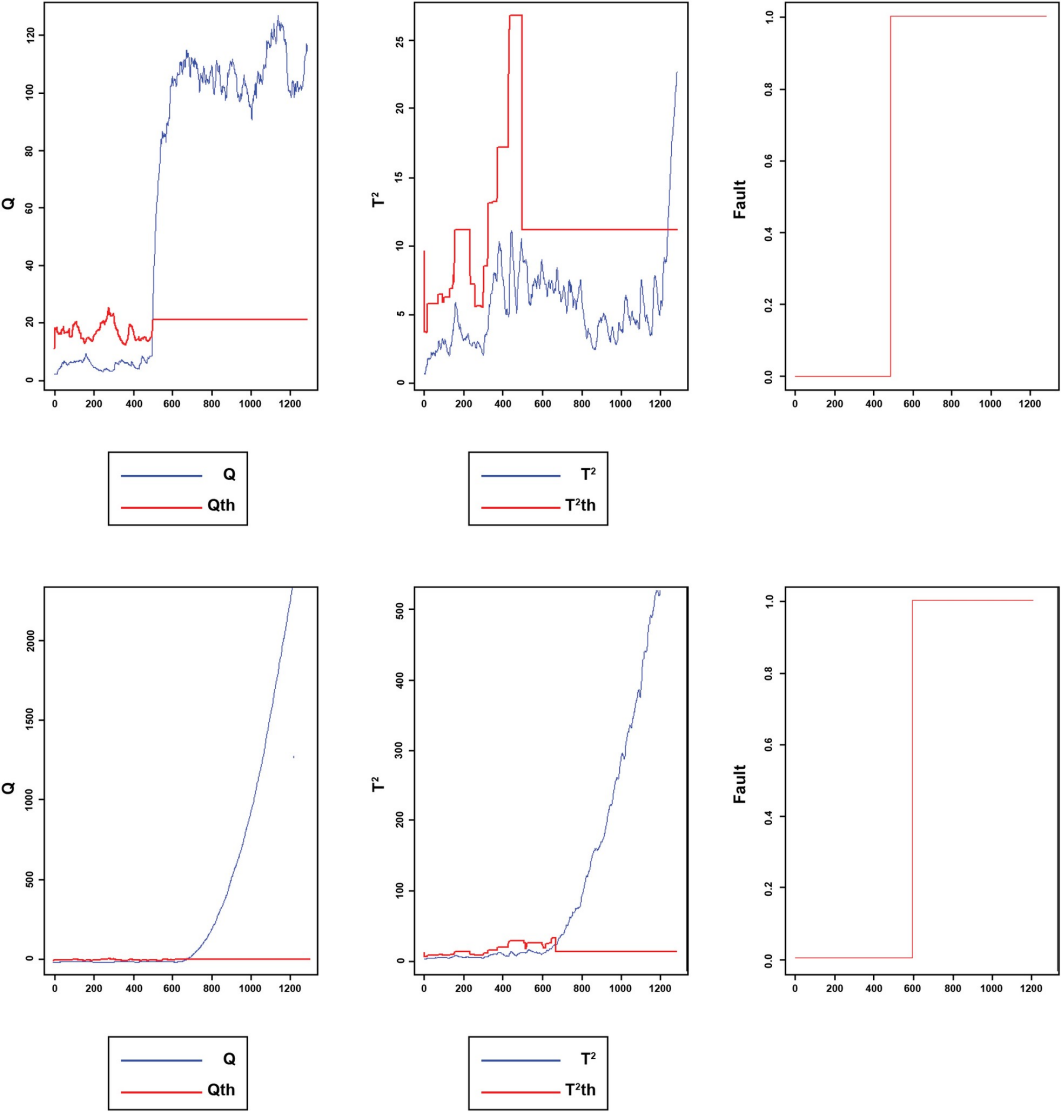
Monitoring performance of MWDPCA for (A) step change (B) slow ramp.

Once the proposed method was applied to the TET data, the number of lags has been selected for each variable, and the developed matrix was constructed in accordance with the lags. Then PCA implemented on data. Two components were retained in accordance with the CPV criterion. Thus, 70.0% of the total variance in the data was explained by these two principal components. The window size was set to 80. The initial and adaptive thresholds were determined at 99% confidence interval. Also, the fixed thresholds were selected experimentally. Fig 18 displays the Q and *T*^2^ of the implemented model for testing fault-free data sets, respectively. Fig 19A and 19B reveal the Q and T2 of the faulty data.

Fig 18 reveals the monitoring performance of MWDPCA for TET data’s normal process.

All the samples are under the control limits. The fault index is 0, meaning that FAR index is zero. Fig 19A illustrates that the process is working in its normal situation, where the observations under the control limits and fault index are zero, and then suddenly, a step change occurs. The fault index switches to one which explains the change in the process situation. Fig 19A indicates that the fault has been detected successfully. For the slow ramp fault, as shown in Fig 19B, MWDPCA can detect the fault well. Table 5 indicates DTD and MDR for faults 1 and 2.

**Table 5:** DTD and MDR for MWDPCA applied on TET data.

	DTD		MDR	
	*Q*	*T*^2^	*Q*	*T*^2^
**Fault 1**	4	5	0.29	0.79
**Fault 2**	18	23	2.45	2.81

## 5. Conclusion

Although, conventional PCA model is a suitable approach for process monitoring, it has not good performance for data with autocorrelation and non-stationary features due to the linearity of this method. As expected, DPCA is an extended PCA model that can decrease the effect of autocorrelation, but like conventional PCA, it has fix thresholds which increases evaluation indicators such as false alarm rate, delaying time detection and missing detection rate. Also, the adaptive PCA models such as RPCA and MWPCA can cope with some kinds of non-stationary data, but due to disregarding the effect of autocorrelation in the data, evaluation indicators do not show good performance, which confuses the operator to make the right decision that may cause undesirable consequences. In this study, we proposed an improved PCA method which is a combination of DPCA and MWPCA method properties which can resolve non-stationary and autocorrelation features, so that the time lag of each variable can differ. This approach was attempted by using the simple structure of DPCA to dominate autocorrelation feature. For this purpose, first, the lag of each variable is calculated and added to the data matrix. Then, by using the property of MWPCA, adaptive thresholds and updating model for each observation is attempted to reduce the effect of non-stationary. The performance of models depended on the reduction rate in some criteria such as False Alarm Rate (FAR), Missed Detection Rate (MDR), and Detection Time Delay (DTD). The proposed method was implemented on TEP data and turbine exhaust temperature as real data. The results of both simulated and real data show that the proposed method has zero FAR in both training and test data set which indicates a good performance in reducing the false alarm rate among other methods. Also, the proposed method was more accurate in MDR and DTD indicators than other methods regardless of the type of faults.

In other words, this method performed better in MDR and DTD indices in 60% of the total faults than other methods. As a result, the proposed method improves process monitoring performance and helps operators make better decisions. However, these approaches needs further improvement to achieve satisfactory monitoring performance. Hence, the use of adaptive thresholds for nonlinear PCA methods could be suggested as future research.

## Supporting information

S1 Fig Monitoring performance of MWDPCA for fault 1. (TIF)

S2 Fig Monitoring performance of MWDPCA for fault 3. (TIF)

S3 Fig Monitoring performance of MWDPCA for fault 4. (TIF)

S4 Fig Monitoring performance of MWDPCA for fault 5. (TIF)

S5 Fig Monitoring performance of MWDPCA for fault 9. (TIF)

S6 Fig Monitoring performance of MWDPCA for fault 10. (TIF)

S7 Fig Monitoring performance of MWDPCA for fault 11. (TIF)

S8 Fig Monitoring performance of MWDPCA for fault 15. (TIF)

S9 Fig Monitoring performance of MWDPCA for fault 16. (TIF)

S10 Fig Monitoring performance of MWDPCA for fault 19. (TIF)

S11 Fig Monitoring performance of MWDPCA for fault 20. (TIF)

## References

[1] Zhang Y, Jiang J. Bibliographical review on reconfigurable fault-tolerant control systems. Annual Reviews in Control. 2008;32(2):229–52. 10.1016/j.arcontrol.2008.03.008.

[2] Chiang LH, Russell EL, Braatz RD. Fault detection and diagnosis in industrial systems: Springer Science & Business Media; 2000.

[3] Russell EL, Chiang LH, Braatz RD. Data-driven methods for fault detection and diagnosis in chemical processes: Springer Science & Business Media; 2012.

[4] Fan C-M, Lu Y-P, editors. A Bayesian framework to integrate knowledge-based and data-driven inference tools for reliable yield diagnoses 2008 Winter Simulation Conference; 2008: IEEE.

[5] Yin S. Data-driven design of fault diagnosis systems: Universität Duisburg-Essen, Fakultät für Ingenieurwissenschaften» Elektrotechnik und Informationstechnik» Automatisierungstechnik und komplexe Systeme; 2012.

[6] Alzghoul A, Backe B, Löfstrand M, Byström A, Liljedahl B. Comparing a knowledge-based and a data-driven method in querying data streams for system fault detection: A hydraulic drive system application. Computers in Industry. 2014;65(8):1126–35. 10.1016/j.compind.2014.06.003.

[7] Sankavaram C, Pattipati B, Kodali A, Pattipati K, Azam M, Kumar S, et al., editors. Model-based and data-driven prognosis of automotive and electronic systems. 2009 IEEE International Conference on Automation Science and Engineering; 2009: IEEE.

[8] Woodall WH, Montgomery DC. Some current directions in the theory and application of statistical process monitoring. Journal of Quality Technology. 2014;46(1):78–94. 10.1080/00224065.2014.11917955

[9] De Ketelaere B, Hubert M, Schmitt E. Overview of PCA-based statistical process-monitoring methods for time-dependent, high-dimensional data. Journal of Quality Technology. 2015;47(4):318–35. 10.1080/00224065.2015.11918137

[10] Rato TJ, Reis MS. Defining the structure of DPCA models and its impact on process monitoring and prediction activities. Chemometrics and Intelligent Laboratory Systems. 2013;125:74–86. 10.1016/j.chemolab.2013.03.009

[11] Rato T, Reis M, Schmitt E, Hubert M, De Ketelaere B. A systematic comparison of PCA‐based statistical process monitoring methods for high‐dimensional, time‐dependent processes. AIChE Journal. 2016;62(5):1478–93. 10.1002/aic.15062

[12] Ku W, Storer RH, Georgakis C. Disturbance detection and isolation by dynamic principal component analysis. Chemometrics and intelligent laboratory systems. 1995;30(1):179–96. 10.1016/0169-7439(95)00076-3

[13] Ammiche M, Kouadri A, Bensmail A. A Modified Moving Window dynamic PCA with Fuzzy Logic Filter and application to fault detection. Chemometrics and Intelligent Laboratory Systems. 2018;177:100–13. 10.1016/j.ces.2018.05.001

[14] Li W, Yue HH, Valle-Cervantes S, Qin SJ. Recursive PCA for adaptive process monitoring. Journal of process control. 2000;10(5):471–86. 10.1016/S0959-1524(00)00022-6

[15] Kruger U, Xie L. Statistical monitoring of complex multivatiate processes: with applications in industrial process control: John Wiley & Sons; 2012.

[16] He XB, Yang YP. Variable MWPCA for adaptive process monitoring. Industrial & Engineering Chemistry Research. 2008;47(2):419–27. 10.1021/ie070712z

[17] Wang X, Kruger U, Irwin GW. Process monitoring approach using fast moving window PCA. Industrial & engineering chemistry research. 2005;44(15):5691–702. 10.1021/ie048873f

[18] Jeng J-C. Adaptive process monitoring using efficient recursive PCA and moving window PCA algorithms. Journal of the Taiwan Institute of Chemical Engineers. 2010;41(4):475–81. 10.1016/j.jtice.2010.03.015.

[19] Jaffel I, Taouali O, Harkat MF, Messaoud H. Moving window KPCA with reduced complexity for nonlinear dynamic process monitoring. ISA transactions. 2016;64:184–92. 10.1016/j.isatra.2016.06.002 27342996

[20] Vanhatalo E, Kulahci M, Bergquist B. On the structure of dynamic principal component analysis used in statistical process monitoring. Chemometrics and intelligent laboratory systems. 2017;167:1–11. 10.1016/j.chemolab.2017.05.016

[21] Huang J, Yan X. Dynamic process fault detection and diagnosis based on dynamic principal component analysis, dynamic independent component analysis and Bayesian inference. Chemometrics and Intelligent Laboratory Systems. 2015;148:115–27. 10.1016/j.chemolab.2015.09.010.

[22] Gao Y, Wang X, Wang Z, Zhao L. Fault detection in time-varying chemical process through incremental principal component analysis. Chemometrics and Intelligent Laboratory Systems. 2016;158:102–16. 10.1016/j.chemolab.2016.07.005.

[23] Jolliffe IT, Cadima J. Principal component analysis: a review and recent developments. Philosophical Transactions of the Royal Society A: Mathematical, Physical and Engineering Sciences. 2016;374(2065):20150202 10.1098/rsta.2015.0202 26953178 PMC4792409

[24] Klyuzhin IS, Fu JF, Hong A, Sacheli M, Shenkov N, Matarazzo M, et al Data-driven, voxel-based analysis of brain PET images: Application of PCA and LASSO methods to visualize and quantify patterns of neurodegeneration. PloS one. 2018;13(11). 10.1371/journal.pone.0206607 PubMed Central PMCID: PMC6218048. 30395576 PMC6218048

[25] Jaffel I, Taouali O, Harkat MF, Messaoud H. Online process monitoring using a new PCMD index. The International Journal of Advanced Manufacturing Technology. 2015;80(5):947–57. 10.1007/s00170-015-7094-2

[26] Valle S, Li W, Qin SJ. Selection of the number of principal components: the variance of the reconstruction error criterion with a comparison to other methods. Industrial & Engineering Chemistry Research. 1999;38(11):4389–401. 10.1021/ie990110i

[27] Li W, Peng M, Wang Q. False alarm reducing in PCA method for sensor fault detection in a nuclear power plant. Annals of Nuclear Energy. 2018;118:131–9. 10.1016/j.anucene.2018.04.012.

[28] Ma J, Amos CI. Principal components analysis of population admixture. PloS one. 2012;7(7). 10.1371/journal.pone.0040115 22808102 PMC3392282

[29] Jackson JE. A user's guide to principal components: John Wiley & Sons; 2005.

[30] Ammiche M. Online Thresholding Techniques for Process Monitoring: University M’Hamed BOUGARA–Boumerdes; 2018.

[31] Ding SX. Data-driven design of fault diagnosis and fault-tolerant control systems: Springer; 2014.

[32] De Ketelaere B, Rato T, Schmitt E, Hubert M. Statistical process monitoring of time-dependent data. Quality Engineering. 2016;28(1):127–42. 10.1080/08982112.2015.1100474

[33] Downs J, Vogel E. A plant-wide industrial process problem control. Comput Chem Eng. 1993;17(3):245–55.

[34] Russell EL, Chiang LH, Braatz RD. Fault detection in industrial processes using canonical variate analysis and dynamic principal component analysis. Chemometrics and intelligent laboratory systems. 2000;51(1):81–93.

[35] Sumana C, Detroja K, Gudi RD. Evaluation of nonlinear scaling and transformation for nonlinear process fault detection. International Journal of Advances in Engineering Sciences and Applied Mathematics. 2012;4(1–2):52–66 10.1007/s12572-012-0060-4

[36] Langston LS, Opdyke G, Dykewood E. Introduction to gas turbines for non-engineers. Global Gas Turbine News. 1997;37(2):1–9.

[37] Ie A. Energy technology perspectives scenarios and strategies to 2050: in support of the G8 Plan of Action. 2006.

