# Supplementary figures and images for: An improved approach for fault detection by simultaneous overcoming of high-dimensionality, autocorrelation, and time-variability
Source: PLoS One. 2020 Dec 17;15(12):e0243146. doi: 10.1371/journal.pone.0243146 (PMC7746307; doi:10.1371/journal.pone.0243146)

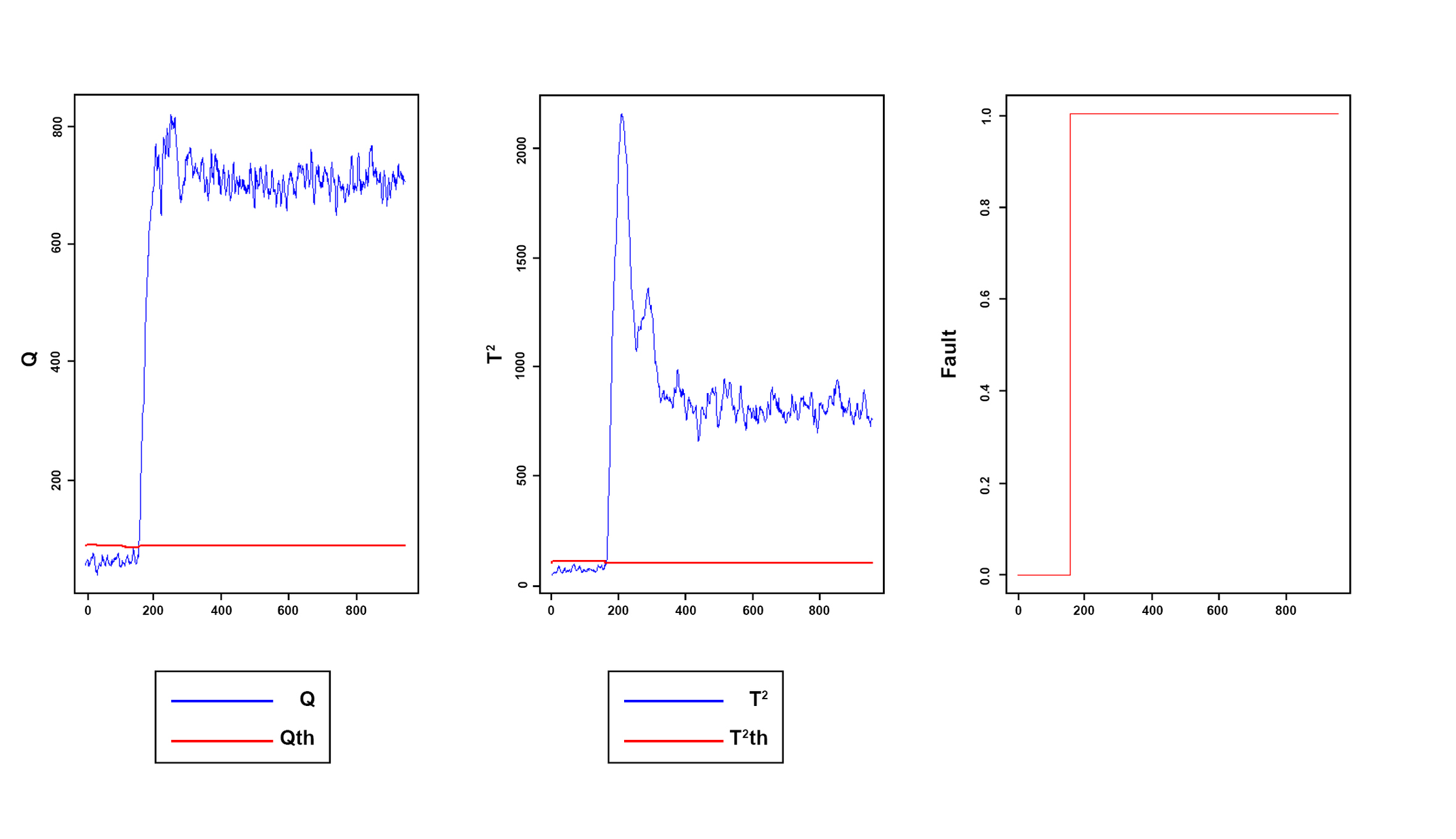

Supplement: S1 Fig — (TIF) [file pone.0243146.s001.tif]

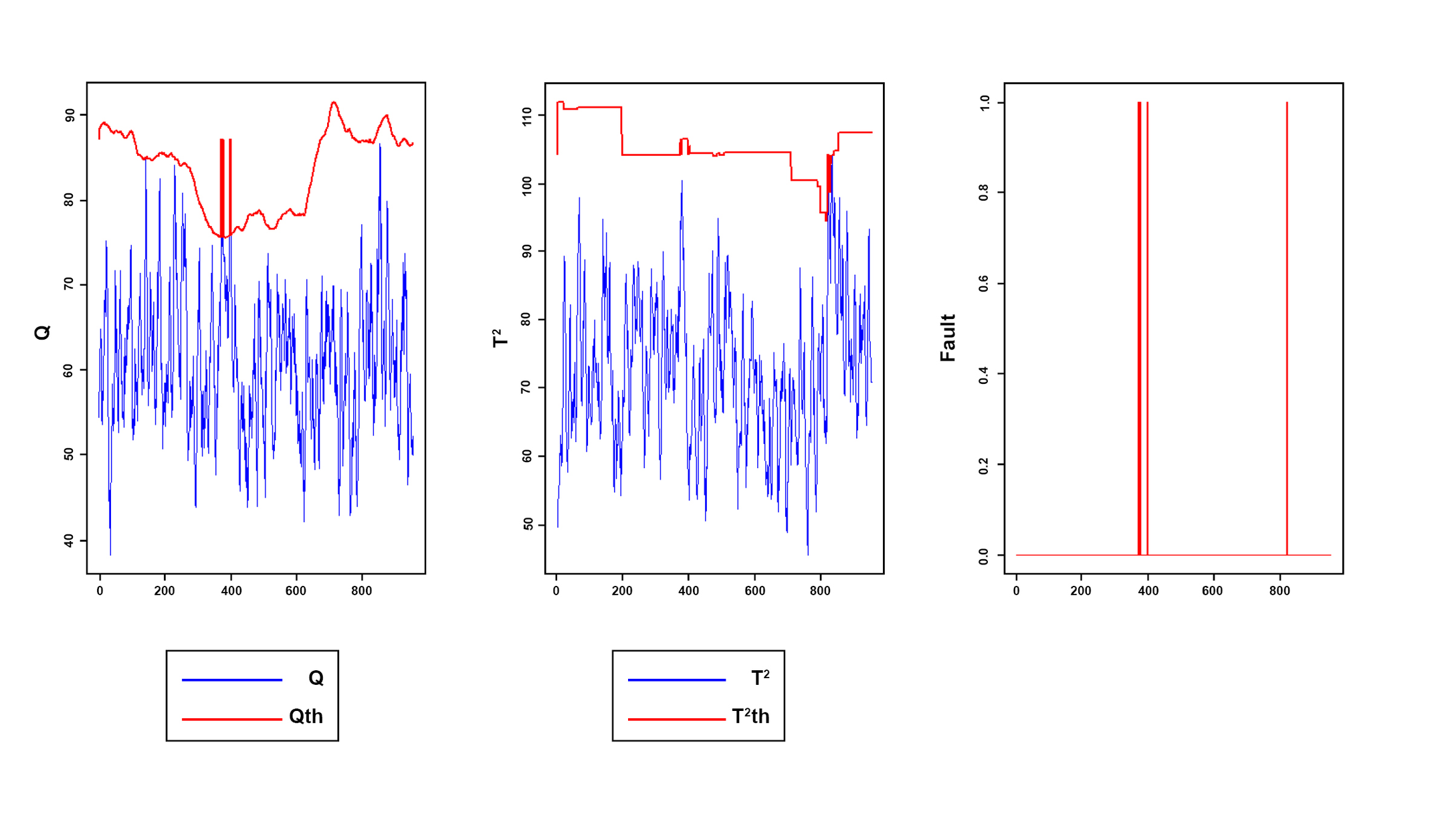

Supplement: S2 Fig — (TIF) [file pone.0243146.s002.tif]

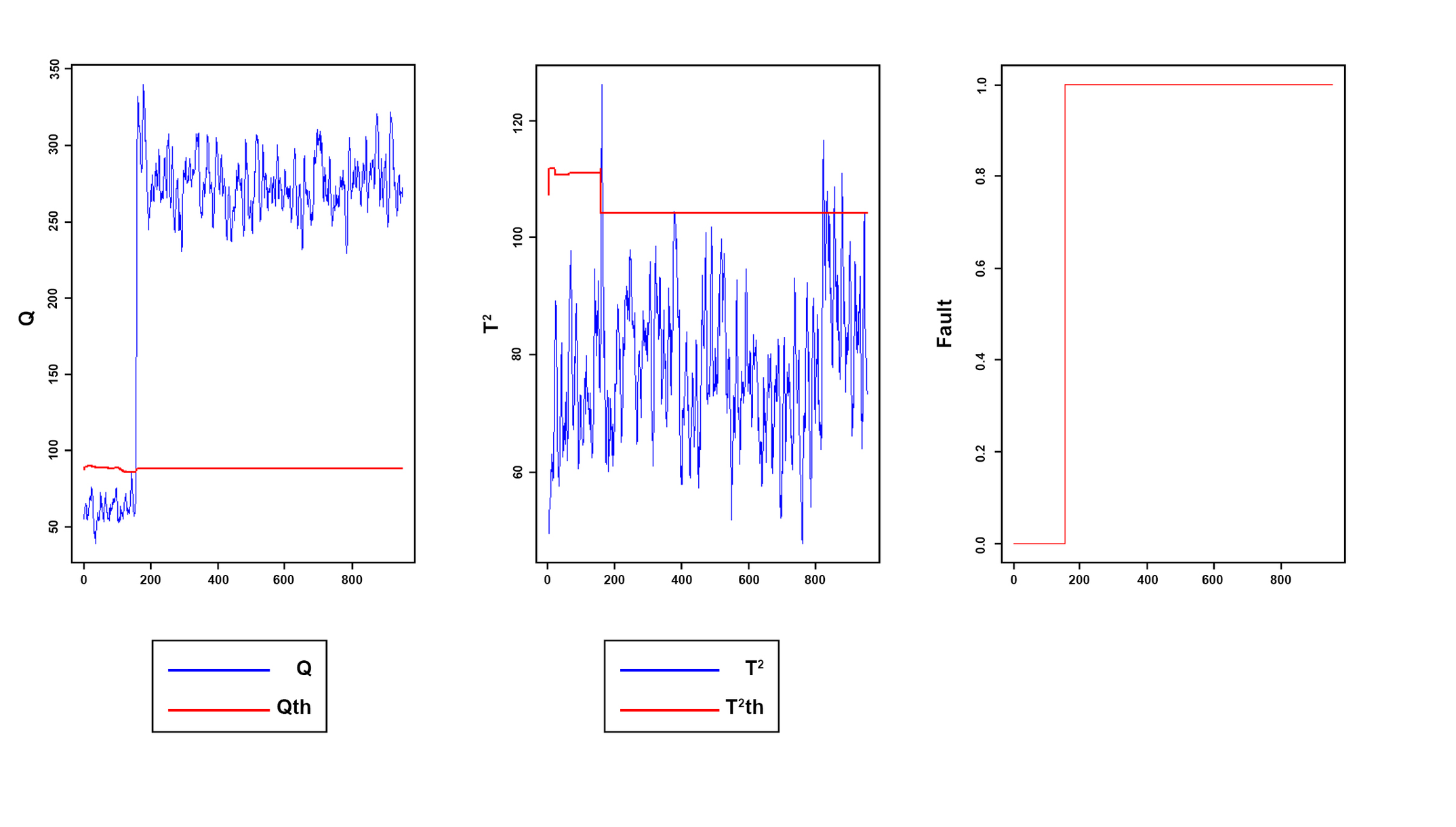

Supplement: S3 Fig — (TIF) [file pone.0243146.s003.tif]

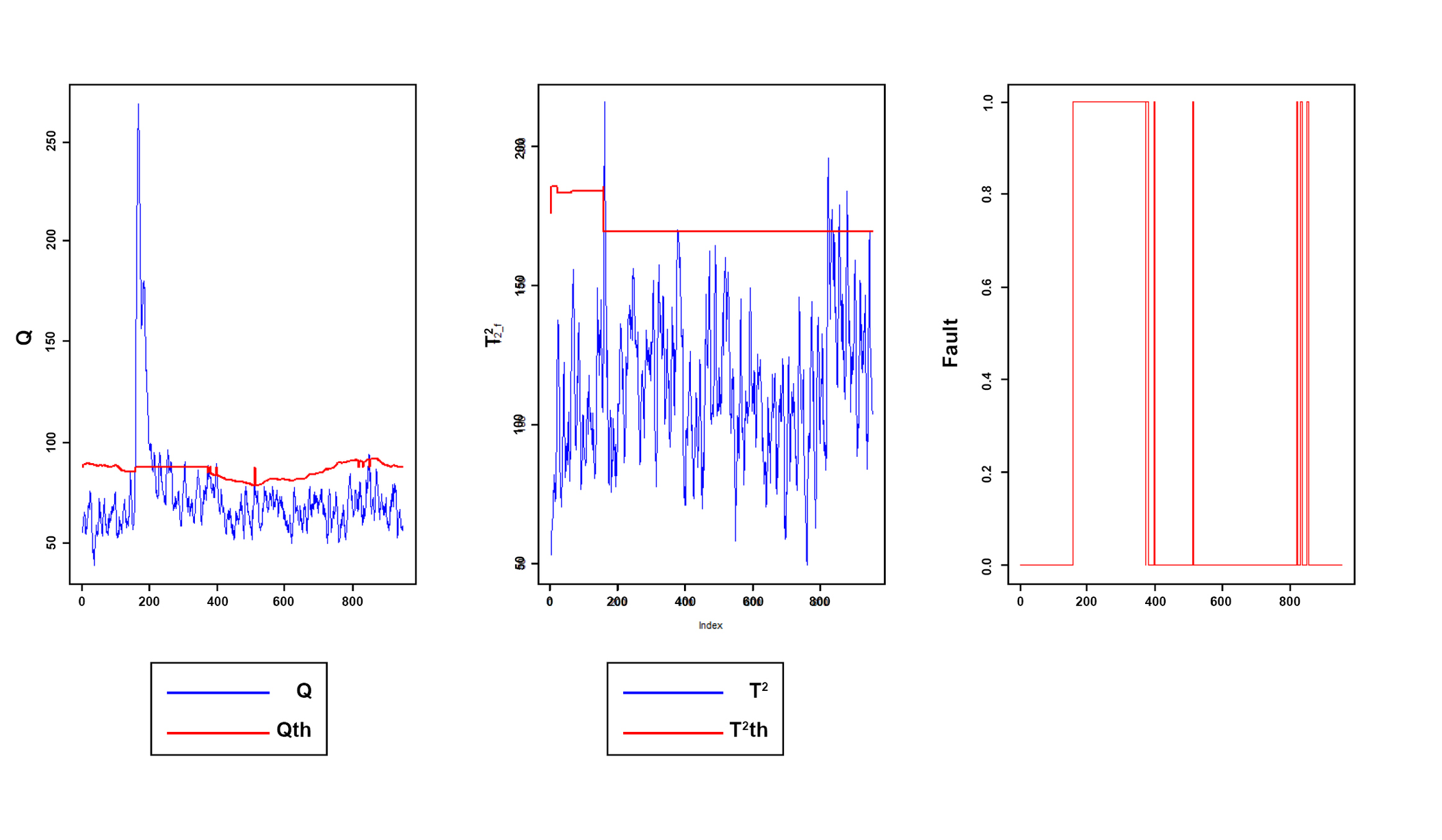

Supplement: S4 Fig — (TIF) [file pone.0243146.s004.tif]

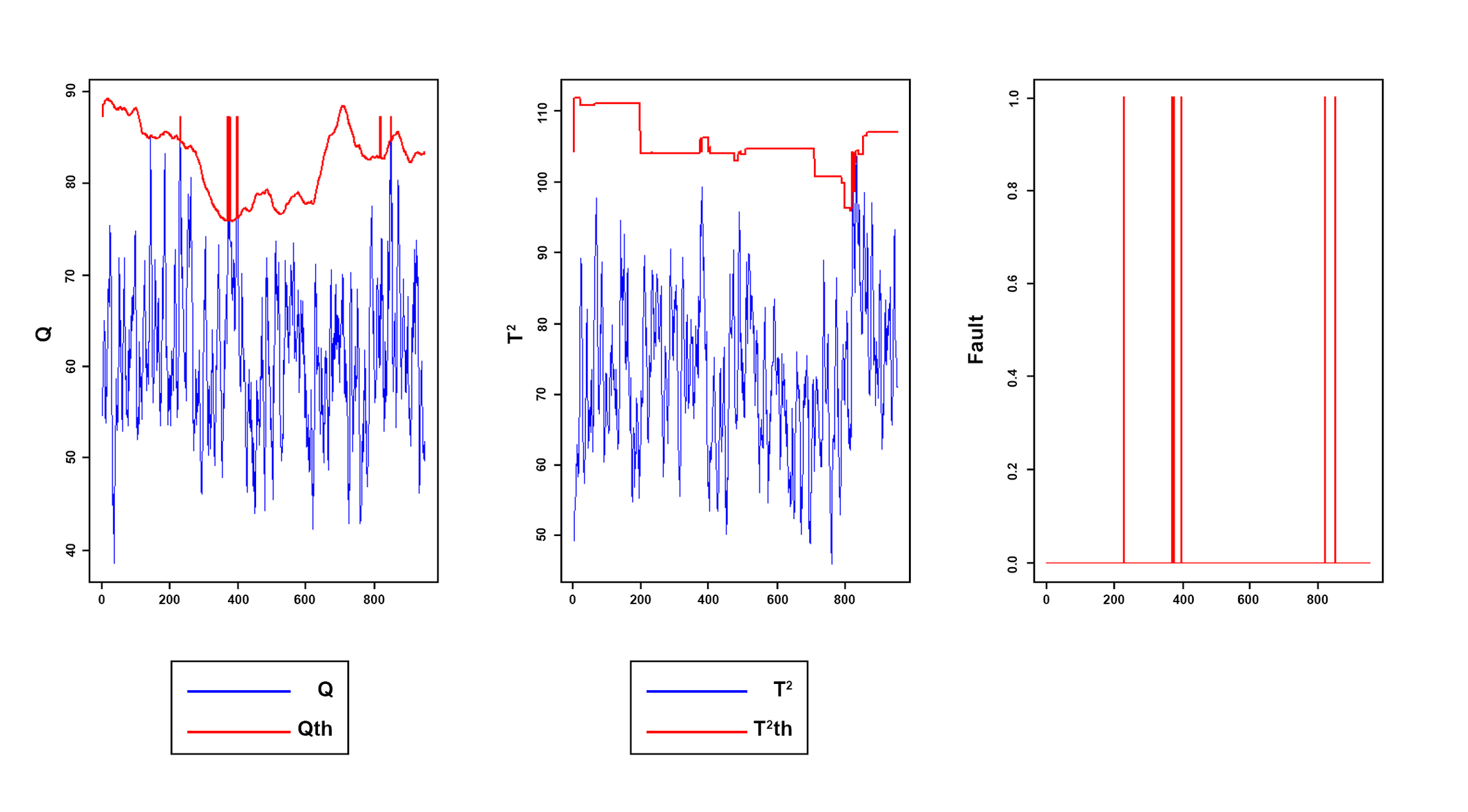

Supplement: S5 Fig — (TIF) [file pone.0243146.s005.tif]

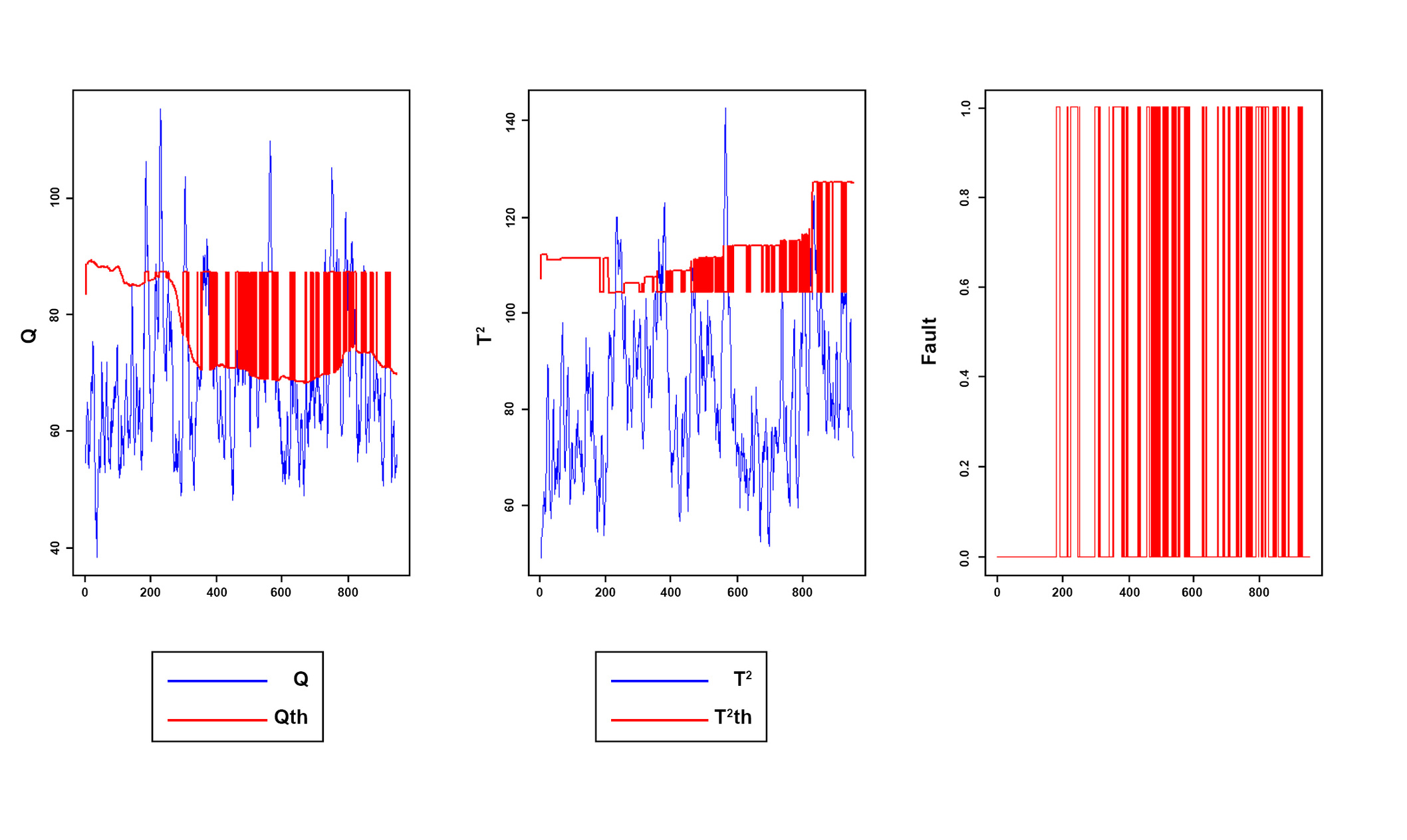

Supplement: S6 Fig — (TIF) [file pone.0243146.s006.tif]

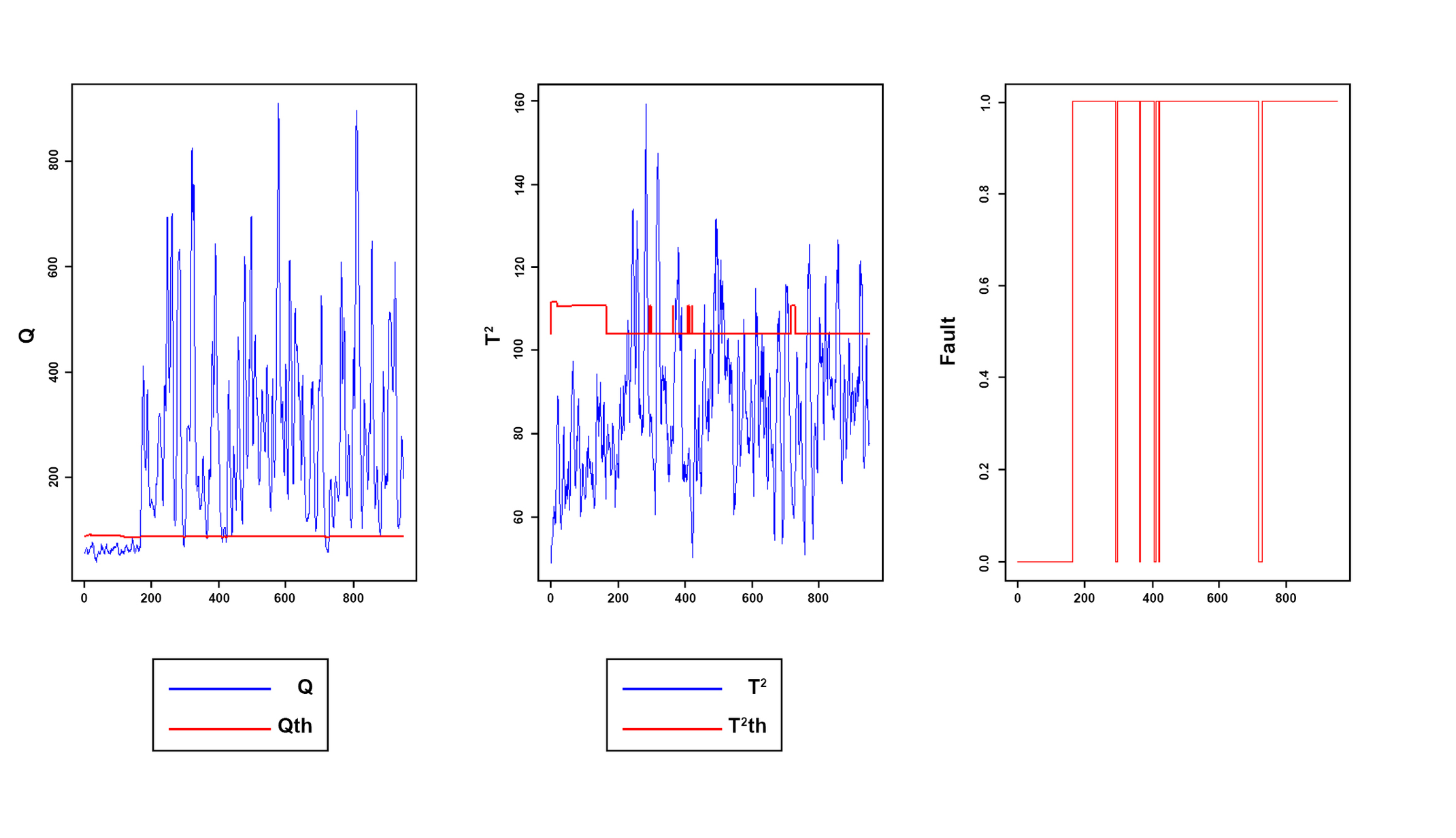

Supplement: S7 Fig — (TIF) [file pone.0243146.s007.tif]

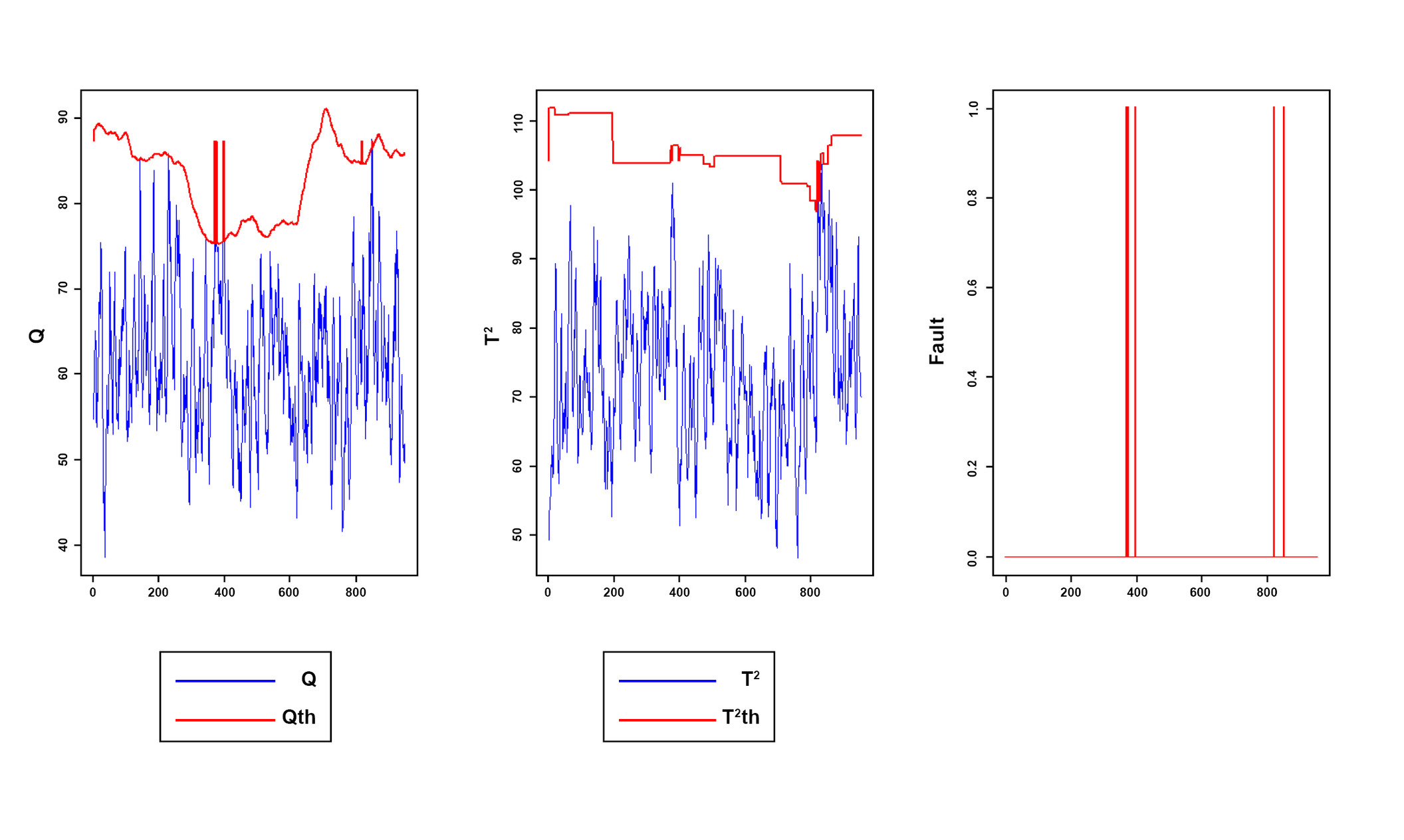

Supplement: S8 Fig — (TIF) [file pone.0243146.s008.tif]

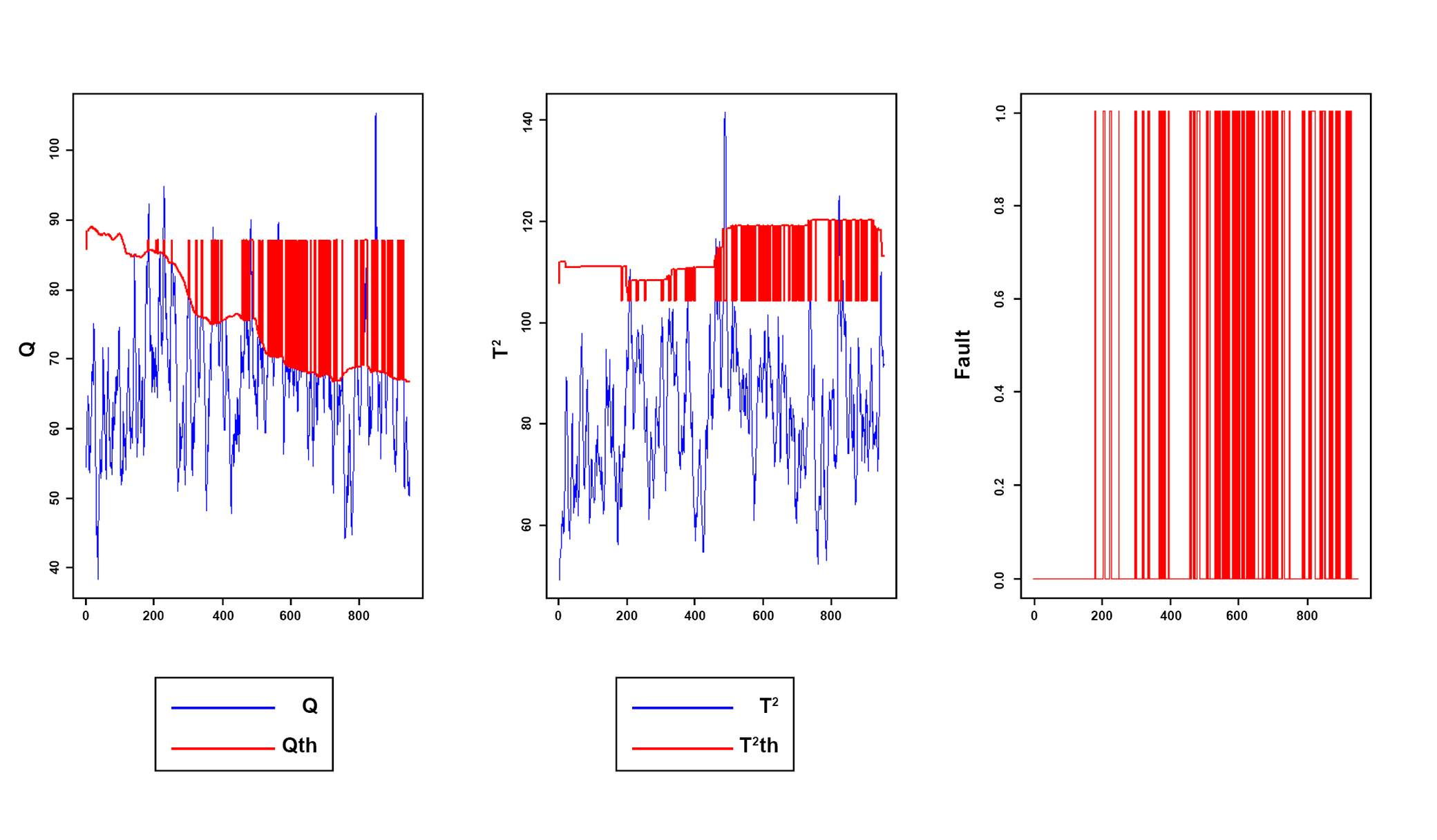

Supplement: S9 Fig — (TIF) [file pone.0243146.s009.tif]

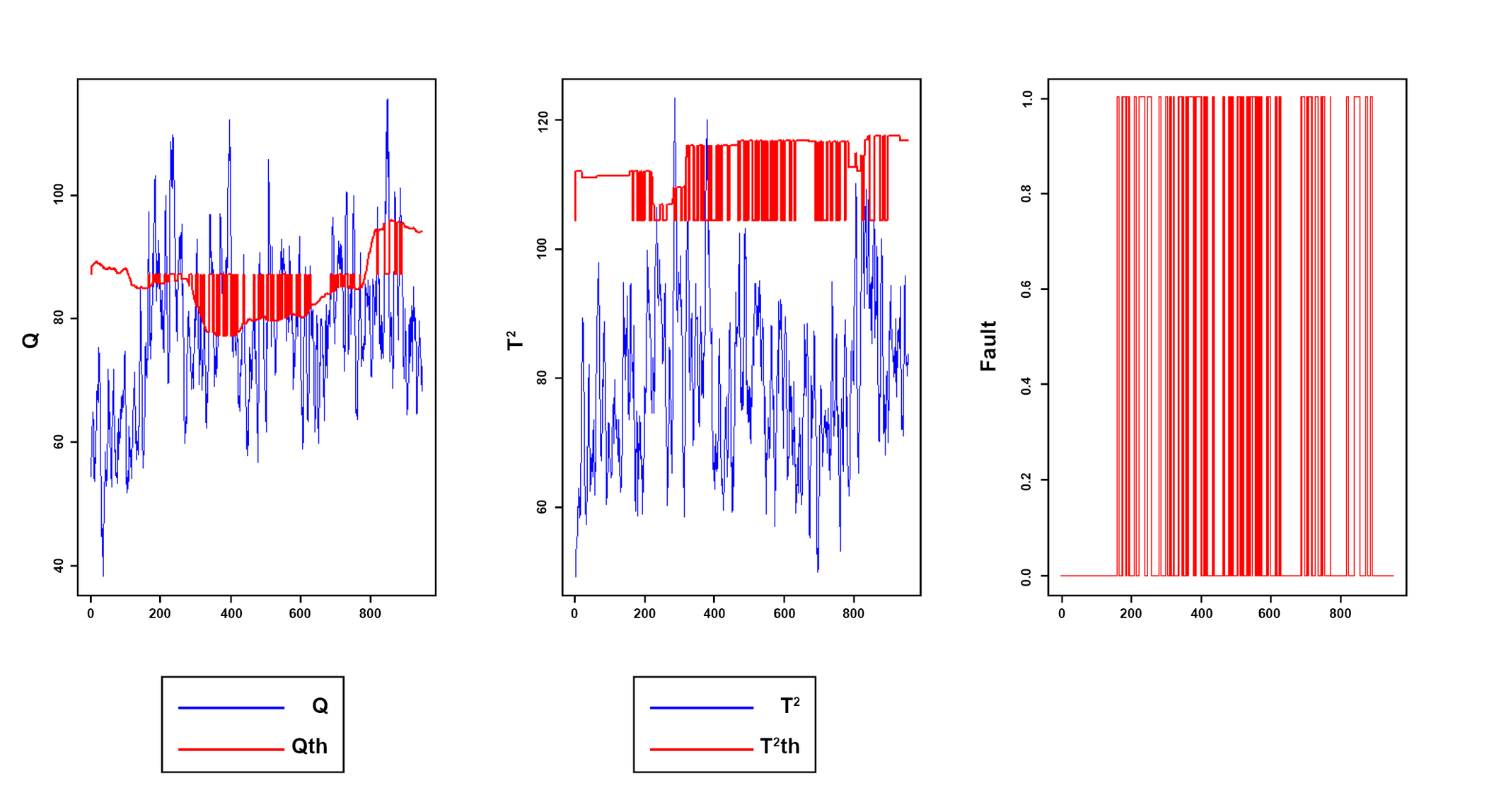

Supplement: S10 Fig — (TIF) [file pone.0243146.s010.tif]

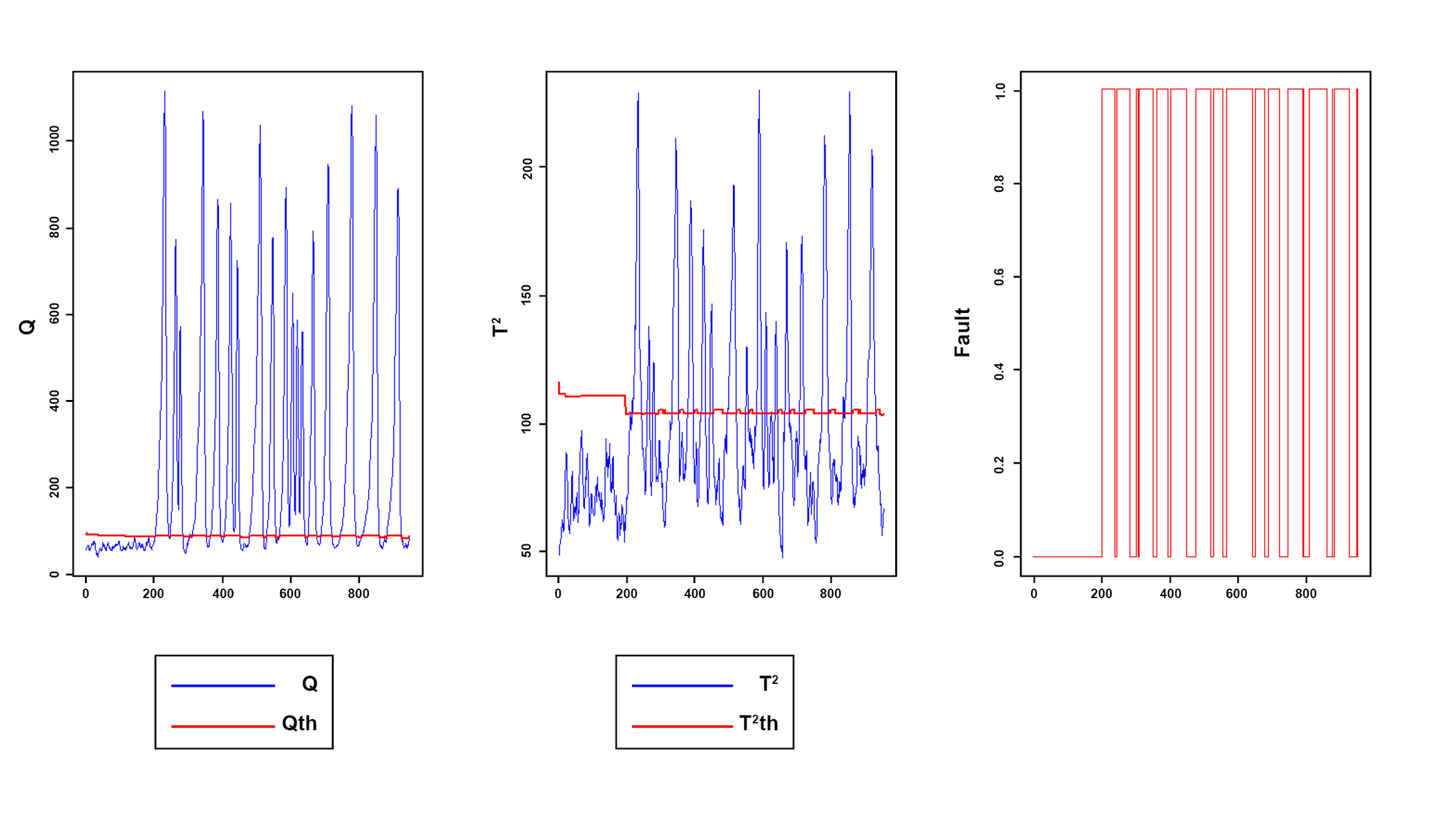

Supplement: S11 Fig — (TIF) [file pone.0243146.s011.tif]
